# Supplementary material for: Socioeconomic status and pulmonary function, transition from childhood to adulthood: cross-sectional results from the polish part of the HAPIEE study
Source: BMJ Open. 2019 Jan 9;9(1):e022638. doi: 10.1136/bmjopen-2018-022638 (PMC6340009; doi:10.1136/bmjopen-2018-022638)
Supplement: Author's manuscript [file bmjopen-2018-022638.draft_revisions.pdf]

BMJ Open is committed to open peer review. As part of this commitment we make the peer review history of every article we publish publicly available.

When an article is published we post the peer reviewers' comments and the authors' responses online. We also post the versions of the paper that were used during peer review. These are the versions that the peer review comments apply to.

The versions of the paper that follow are the versions that were submitted during the peer review process. They are not the versions of record or the final published versions. They should not be cited or distributed as the published version of this manuscript.

BMJ Open is an open access journal and the full, final, typeset and author-corrected version of record of the manuscript is available on our site with no access controls, subscription charges or pay-per-view fees (http://bmjopen.bmj.com).

If you have any questions on BMJ Open's open peer review process please email info.bmjopen@bmj.com

BMJ Open

Socioeconomic status and pulmonary function, transition from childhood to adulthood: cross-sectional results from the Polish part of the HAPIEE study.

Journal:	*BMJ Open*
Manuscript ID	bmjopen-2018-022638
Article Type:	Research
Date Submitted by the Author:	09-Mar-2018

Complete List of Authors:	Polak, Maciej; Jagiellonian University Medical College, Krakow, Poland, Chair of Epidemiology and Population Studies, Faculty of Health Sciences, Szafraniec, Krystyna; Jagiellonian University Medical College, Krakow, Poland, Chair of Epidemiology and Population Studies, Faculty of Health Sciences<br>Kozela, Magdalena ; Jagiellonian University Medical College, Krakow, Poland, Chair of Epidemiology and Population Studies, Faculty of Health Sciences,<br>Wolfshaut-Wolak, Renata; Jagiellonian University Medical College, Krakow, Poland, Chair of Epidemiology and Population Studies, Faculty of Health Sciences<br>Bobak, Martin; University College London, Epidemiology and Public Health<br>Pająk, Andrzej; Jagiellonian University Medical College, Krakow, Poland, Chair of Epidemiology and Population Studies, Faculty of Health Sciences

Keywords:	SES, Lung function, Social mobility

SCHOLARONE™ Manuscripts

For peer review only - http://bmjopen.bmj.com/site/about/guidelines.xhtml

Title:
Socioeconomic status and pulmonary function, transition from childhood to adulthood: cross-sectional results from the Polish part of the HAPIEE study

Maciej Polak, Krystyna Szafraniec, Magdalena Kozela, Renata Wolfshaut-Wolak, Martin Bobak, Andrzej Pająk

Affiliation: Chair of Epidemiology and Population Studies, Institute of Public Health, Jagiellonian University Medical College

Corresponding author:

Maciej Polak

Chair of Epidemiology and Population Studies
Faculty of Health Science
Jagiellonian University Medical College
31-531 Kraków, ul. Grzegórzecka 20
phone: +48 12 43 32 837
e-mail: maciej.1.polak@uj.edu.pl

For peer review only

For peer review only - http://bmjopen.bmj.com/site/about/guidelines.xhtml

Abstract

Objective

Previous studies reported inverse associations between socioeconomic status (SES) and lung function but less is known whether pulmonary function is affected by SES changes.

We aimed to describe the relation of changes of SES between childhood and adulthood with pulmonary function.

Design

Cross-sectional study.

Participants

The study sample included 4104 men and women, aged 45–69 years, residents of Krakow, participating in the Polish part of the HAPIEE Project

Main outcome

Forced expiratory volume (FEV_1) and forced vital capacity (FVC) were assessed by the standardized spirometry procedure. Participants were classified into the 3 categories of SES (low, moderate, or high) based on the information on parent's education, housing standard during childhood, own education, professional activity, household amenities, and financial status.

Results

The adjusted difference in mean FVC between persons with low and high adulthood SES was 100 ml (p=0.005) in men and 100 ml (p<0.001) in women; the differences in mean FEV_1 were 103 ml (p<0.001) and 80 ml (p<0.001), respectively. Participants who changed SES during life course from low at childhood to moderate or high at adulthood and those who had moderate or high SES at both childhood and adulthood had significantly higher FEV_1 and FVC compared to those who had always low SES or SES changed to low.

Conclusions

Low SES over life-course was associated with the lowest lung function. Downward social mobility was associated with poorer pulmonary function while upward mobility or life-course moderate or high SES were associated with better pulmonary function.

Strengths and limitations of this study.

Strengths

- Addresses the association between social mobility and pulmonary function in adults.
- This is the first study of the association between SES and pulmonary function in Eastern Europe, a region undergoing profound social and political transformation after the fall of the communism
- Multidimensional nature of SES was captured by application of cluster analysis

Limitation

- The cross-sectional study design does not allow address the causality of the observed relations
- Retrospective collection of information on childhood SES at age 10 could be subject to recall bias

For peer review only

Introduction

Socioeconomic status (SES) is one of the main predictors of health[1-3]. Earlier studies have clearly showed a gradient effect for the cardiovascular disease (CVD) mortality and morbidity as well as the prevalence of the CVD risk factors, regardless of the health measures and SES indicators used[4-6]. At an individual level, the common indicators of SES were income, education, and occupation. However, recent studies showed the relation between health and new index of SES that expresses the "subjective" perceptions of an individual's placement in the social hierarchy[7,8].

Several social, psychological, and biological mechanisms have been proposed to explain the association of SES to health including current hypothesis on exerting adverse health consequences via allostatic load[9-12]. On the other hand, there is also some evidence for reverse hypothesis on health to SES relationship: "social drift" links poor health to a subsequent downward shift in the social class[13,14].

Recently, the life course approach to how SES effects health has been increasingly applied, because it allows going beyond independent effects of childhood and adulthood SES exposure for disease development[15-17]. Whilst the life course approach has already been implemented to study the effects of SES on CVD[18], obesity[5], hypertension[19], and diabetes[6], it has been applied less frequently to respiratory diseases[20,21].

Lung function is a long-term predictor of morbidity and mortality from a range of diseases including non-respiratory conditions[22,23]. Studies have demonstrated the association of a decrease in lung function not only with increasing age, but also with low SES[24]. However, little is known how the changes in SES patterns over the influence the respiratory health. None of the research from Central and Eastern Europe (CEE) examined this association and hence this topic becomes challenging. Especially because of political transformation of 1989/90 occurring in this region, followed by rapid social and economic changes, resulted in widening income inequalities and changes in health behavior. Additionally, most of middle-aged people's life crossed over that time period resulting in some phenomena that childhood SES was well established before and adulthood SES have been developing after the fall of socialism.

The aim of the present study was to describe the relation of changes of SES between childhood and adulthood with pulmonary function.

For peer review only - http://bmjopen.bmj.com/site/about/guidelines.xhtml

Methods

Study design and data collection

The analysis was performed using data from the Polish part of the HAPIEE (*Health, Alcohol and Psychosocial Factors In Eastern Europe*) study. The design of this study has been described in detail elsewhere[25]. Briefly, the HAPIEE project involved random samples of residents of Krakow aged between 45 and 69. Trained nurses interviewed the respondents at their homes. Information about age, education, marital status, employment, health status (asthma or history of CVD), and health behavior, i.e., smoking and physical activity was obtained using a standard questionnaire. Then all participants were invited to a clinic for anthropometric and blood pressure measurements followed by blood collection for biochemical tests and for performing spirometry.

Patient and Public Involvement statement

Participants were not involved in the design of this study.

Pulmonary function assessment

Forced vital capacity (FVC) and one-second forced expiratory volume (FEV_1) were measured by a trained technician in accordance with a standardized protocol[26] using Micro-Medical Microplus spirometer. Participants were asked to make at least three maximal expiratory measures. If the quality of the spirometry was not satisfactory, the maneuver was repeated until the best quality was obtained. The highest value of FVC and the highest value of FEV_1 were chosen from these measurements for which the repeatability criteria were met. The quality of all tests was reviewed by a pulmonologist. Criteria for a satisfactory repeatability were based on the European Respiratory Society recommendations[26]. Exclusion criteria for the spirometry included any chest surgery during last three months, any physician-diagnosed serious cardiac condition (stroke, myocardial infarction), and recent respiratory infections.

Socioeconomic Status

Childhood SES was assessed using the level of education of both parents (incomplete primary or no formal education, primary, vocational, secondary, or university) and

For peer review only - http://bmjopen.bmj.com/site/about/guidelines.xhtml

information about housing standard at the age of about 10 (cold tap water, hot tap water, radio, fridge, own kitchen, and own toilet), all summed up into an index measuring the amenities in childhood [range 0–6].

Adulthood SES was measured by five items: the study of participant's educational attainment, professional activity, household amenities, and current financial situation. Participant's education was assessed by questioning his/her highest level of education completed. Current professional position was based on the employment status and categorized into: an owner of a company, employed, retired, or unemployed. Index of household amenities was constructed by summing up the number of valuable items that a participant had in his household (eg microwave, washing machine) [range 0–12]. Current financial status was assessed by two questions: "How often does it happen not to have enough money for food which you and your family need?" and "Do you have any difficulties with paying bills?". The possible answers were the following: all the time, often, sometimes, rarely, or never.

Two-stage clustering algorithm was applied to classify participants into homogeneous groups of childhood SES and adulthood SES. We allowed the process to identify the dimensions of SES without constraint to any particular number of factors, in order to capture the natural complexity of SES. Three groups of childhood SES were generated, assessing reasonable evidence of the cluster structure (silhouette coefficient s(i)=0.51). Participants who had poor housing conditions (low value of childhood amenities index) and their parents had no formal education or had at the most primary school were classified by the algorithm as a low SES group. The middle SES group comprised of subjects characterized by medium housing condition in childhood and secondary education completed by at least one parent of the participants. High value of childhood amenities index and parent's secondary or university education classified a participant into the high childhood SES group. Similarly, three groups of adult SES were identified (s(i)=0.55). Participants who had a university education, were employed, and had a high index of amenities were classified by the cluster algorithm as having high SES in adulthood. On the other hand, participants with low educational achievements, unemployed, being on retirement pension, or reporting lack of money for food or paying bills have been classified into low SES group. The middle SES group comprised of participants characterized mainly by secondary education (however, 44% had a primary of education), half-and-half working and retired persons having no problem with money for food and paying bills regularly (**table 1**).

For peer review only - http://bmjopen.bmj.com/site/about/guidelines.xhtml

Table 1. Definition of the socioeconomic status

Socioeconomic status		Descriptive statistics	Cluster description
Childhood	Low	n=1632<br>Age (years): 58.9<br>Female (%):51	- Primary parent's education<br>- Low value of amenities index in childhood
	Middle	n=1463<br>Age (years): 58.4<br>Female (%): 51	- Primary or secondary parent's education<br>- Low or middle value of amenities index in childhood
	High	n=881<br>Age (years): 56.0<br>Female (%): 50	- Secondary or university parent's education<br>- High value of amenities index in childhood
Adulthood	Low	n=1313<br>Age (years): 57.8<br>Female (%): 58	- Primary or secondary education<br>- Often or sometimes do not have enough money for food or have problems with paying bills<br>- Unemployed or pensioner
	Middle	n=1588 Age (years): 58.9<br>Female (%): 49	- Primary or secondary education<br>- Rarely or never don't have enough money for food or have problems with paying bills
	High	n=1101<br>Age (years): 57.1<br>Female (%): 44	- University education<br>- Employed or owner of company<br>- High value of amenities index

For peer review only

The cumulative SES score was calculated by summing the childhood and adulthood SES evaluations. The final cumulative SES score was further categorized as low (score=0–1), medium (score=2–3), and high (score=4).

Transition from childhood to adulthood

Having separate estimates for early childhood SES and adult SES, it allowed each participant to be allocated to one of the following four separate group of SES changes: participants who were classified in the low subgroups in both time points were categorized as "always low". Those who were classified in the low SES in childhood, but in adulthood in middle or high

For peer review only - http://bmjopen.bmj.com/site/about/guidelines.xhtml

were categorized as ‘change from low to moderate or high”. Similarly, those classified in middle or high SES in childhood and low in adulthood were categorized as “change from moderate or high to low”. The categories moderate and high SES were combined due to similar differences in pulmonary function of participants who changed SES from low to moderate and from low to high SES. Finally, those classified in both life stages in the middle or high SES were categorized as “always moderate or high”.

Statistical analysis

Descriptive statistics stratified by sex were presented as means and standard deviations for pulmonary function parameters. Two stage cluster analysis was used to determine homogenous subgroups of childhood SES and adult SES. In the modeling process, the hierarchical clustering method and the log-likelihood distance measure between clusters were used. The validation of consistency within clusters of data was based on silhouette coefficient s(i). Values above 0.5 are recognized as either reasonable or strong evidence of cluster structure (details has been briefly described in[27]).

To compare FVC and FEV_1, the distribution by SES in childhood, in adulthood, and in transition of SES between these two periods, a set of analysis of covariance were performed separately for men and women. The final models were adjusted for age, height, BMI, smoking, and prevalence of respiratory diseases. Data was analyzed using IBM® SPSS® ver.22. Significance level was accepted at alpha=0.05.

For peer review only

For peer review only - http://bmjopen.bmj.com/site/about/guidelines.xhtml

Results

Table 2. Characteristics of the studied sample by sex

	Male	n=2020	Female	n=2084	p
Age (mean, sd)	57.9	6.86	57.2	6.962	0.6[A]
FEV_1 [L](mean, sd)	3.15	0.659	2.27	0.473	<0.001[A]
FVC [L] (mean sd)	3.81	0.717	2.73	5.280	<0.001[A]
BMI (mean, sd)	28.10	4.130	28.47	5.040	0.016[A]
Smoking status					
Current smoker	666	33.1	539	25.9	
Ex-smoker	755	37.5	419	20.2	<0.001[B]
Never smoker	592	29.4	1121	53.9	
Respiratory diseases (%)	266	13.4	285	13.8	0.6[B]
Adulthood SES					
Low	548	27.1	765	36.7	
Middle	804	39.8	784	37.6	<0.001[B]
High	614	30.4	487	23.9	
Childhood SES					
Low	799	40.7	833	41.3	
Middle	723	36.9	740	36.7	0.9[B]
High	439	22.4	442	21.9	
Transitions SES					
Always low	225	11.7	314	15.9	
Change from moderate or high to low	308	16.1	424	21.5	
Change from low to moderate or high	557	29.1	503	25.5	<0.001[B]
Always moderate or high	825	43.1	732	37.1	
Cumulative SES score					
Low	833	43.5	996	50.5	
Middle	729	43.3	769	39	<0.001[B]
High	253	13.2	208	10.5	

A–p value for t-test
B–p value for chi-square test

Out of 4840 participants for whom spirometry measurements were performed, 4104 (84.8%) participants provided valid spirometry data and were included in this analysis. There was no difference in age, BMI, smoking, and SES level in childhood between analyzed group and excluded participants. But, the latter group had a higher prevalence of respiratory diseases and higher percentage (by 7%) of low SES category in adulthood. The characteristics of the study

For peer review only - http://bmjopen.bmj.com/site/about/guidelines.xhtml

sample are presented in **table 2**. The mean age was 55.6 (SD=6.92) years and it did not differ significantly by sex. Mean FEV_1 for the whole sample was 2.70 L (95% CI 2.68 to 2.73), men had higher mean FEV_1 by 0.88 L, compared to women. Mean FVC was 3.25 L (95% CI 3.22 to 3.28), difference between men and women in mean FVC was statistically significant and was equal to 1.08 L. Distribution of SES indicators (adulthood, cumulative, and transition) differed between sexes. Low SES in adult was observed in 27% of men and 37% of women ($p<0.001$). No differences in the distribution of childhood SES emerged from sex groups.

Tables 3 and **4** show the adjusted means for age as well as all covariate means of FEV_1 and FVC by sex, SES, and SES transition categories. Age adjusted means of FEV_1 and FVC were associated with childhood (in women), adulthood, and cumulative SES score with significant gradient in men and women. Compared to high SES, men in the low or middle SES had significantly worse lung function (differences ranging from 0.20 L to 0.27 L in FEV_1 and 0.10 L to 0.26 L in FVC). Results were similar but less pronounced in women (differences from 0.07 L to 0.16 L in case of FEV_1 and 0.18 L to 0.25 L in FVC). Controlling for age, height, BMI, smoking, and prevalence of respiratory diseases resulted in some attenuation of the estimates, but the pattern and significance of the association remained, except for the relation between childhood SES and FVC, as well as cumulative SES and FEV_1 and FVC in men.

For peer review only

For peer review only - http://bmjopen.bmj.com/site/about/guidelines.xhtml

Table 3. **Means of FEV_1 [L] by category of childhood, adulthood SES, transition SES, and cumulative assessment of SES**

	Male				Female			
	model A		model B		model A		model B	
Adulthood SES	mean	95% CI	mean	95% CI	men	95% CI	mean	95% CI
Low	3.01	(2.96-3.06)	2.9	(2.85-2.94)	2.22	(2.19-2.25)	2.12	(2.09-2.15)
Middle	3.16	(3.12-3.20)	2.99	(2.95-3.03)	2.27	(2.24-2.30)	2.15	(2.12-2.18)
High	3.28	(3.24-3.32)	3.03	(2.98-3.07)	2.36	(2.33-2.40)	2.2	(2.16-2.24)
p for trend	<0.001		<0.001		<0.001		0.02	
Childhood SES	mean	95% CI	mean	95% CI	mean	95% CI	mean	95% CI
Low	3.15	(3.11-3.19)	3.00	(2.95-3.04)	2.27	(2.24-2.29)	2.16	(2.13-2.19)
Middle	3.18	(3.08-3.27)	2.95	(2.90-2.99)	2.25	(2.22-2.28)	2.12	(2.09-2.16)
High	3.19	(3.14-3.25)	2.94	(2.89-3.00)	2.34	(2.30-2.37)	2.19	(2.15-2.23)
p for trend	0.1		0.1		0.001		0.011	
Cumulative SES score	mean	95% CI	mean	95% CI	mean	95% CI	mean	95% CI
Low	3.09	(3.05-3.13)	2.95	(2.91-3.00)	2.24	(2.18-2.24)	2.14	(2.11-2.17)
Middle	3.18	(3.14-3.22)	2.97	(2.93-3.01)	2.29	(2.26-2.32)	2.15	(2.12-2.18)
High	3.29	(3.22-3.36)	2.99	(2.92-3.06)	2.4	(2.35-2.46)	2.23	(2.17-2.28)
p for trend	<0.001		0.6		<0.001		0.009	
Change in SES	mean	95% CI	mean	95% CI	mean	95% CI	mean	95% CI
Always low	2.98	(2.90-3.05)	2.91	(2.84-2.98)	2.21	(2.17-2.26)	2.14	(2.10-2.19)
Change from moderate or high to low	3.03	(2.97-3.10)	2.88	(2.82-2.94)	2.23	(2.19-2.27)	2.13	(2.09-2.17)
Change from low to moderate or high	3.21	(3.16-3.26)	3.03	(2.98-3.08)	2.3	(2.27-2.34)	2.19	(2.15-2.23)
Always moderate or high	3.21	(3.17-3.24)	3.04	(2.98-3.07)	2.31	(2.28-2.34)	2.21	(2.18-2.24)
p for trend	<0.001		<0.001		<0.001		0.048	

model A—adjusted for age

model B—adjusted for age, height, BMI, smoking status and respiratory disease.

For peer review only

For peer review only - http://bmjopen.bmj.com/site/about/guidelines.xhtml

Table 4. **Means of FVC [L] in category of childhood, adulthood, and transition SES and cumulative assessment of SES**

	Male				Female			
	model A		model B		model A		model B	
Adulthood SES	mean	95% CI	mean	95% CI	mean	95% CI	mean	95% CI
Low	3.66	(3.61-3.72)	3.61	(3.55-3.67)	2.67	(2.64-2.71)	2.63	(2.59-3.66)
Middle	3.83	(3.78-3.88)	3.73	(3.68-3.78)	2.71	(2.67-2.74)	2.65	(2.61-2.69)
High	3.92	(3.86-3.97)	3.71	(3.65-3.76)	2.85	(2.81-2.89)	2.73	(2.68-2.77)
p for trend	<0.001		0.005			<0.001		<0.001
Childhood SES	mean	95% CI	mean	95% CI	mean	95% CI	mean	95% CI
Low	3.79	(3.74-3.83)	3.71	(3.66-3.76)	2,69	(2.66-2.72)	2.642	(2.61-2.68)
Middle	3.77	(3.72-3.82)	3.66	(3.60-3.71)	2.71	(2.68-2.75)	2.635	(2.60-2.67)
High	3.89	(3.83-3.96)	3.69	(3.62-3.75)	2.84	(2.79-2.88)	2.725	(2.68-2.77)
p for trend		0.001		0.1		<0.001		0.001
Cumulative SES score	mean	95% CI	mean	95% CI	mean	95% CI	mean	95% CI
Low	3.73	(3.67-3.78)	3.67	(3.62-3.72)	2.67	(2.64-2.70)	2.63	(2.60-2.67)
Middle	3.84	(3.80-3.89)	3.69	(3.64-3.74)	2.76	(2.72-2.79)	2.66	(2.62-2.70)
High	3.93	(3.84-4.02)	3.67	(3.58-3.75)	2.92	(2.85-2.98)	2.78	(2.71-2.84)
p for trend		<0.001		0.7		<0.001		<0.01
Change in SES	mean	95% CI	mean	95% CI	mean	95% CI	mean	95% CI
Always low	3.59	(3.50-3.68)	3.6	(3.52-3.67)	2.64	(2.58-2.69)	2.62	(2.57-2.67)
Change from moderate or high to low	3.72	(3.64-3.80)	3.63	(3.56-3.70)	2.71	(2.66-2.75)	2.64	(2.59-2.68)
Change from low to moderate or high	3.87	(3.81-3.92)	3.77	(3.71-3.83)	2.72	(2.68-2.76)	2.67	(2.63-2.72)
Always moderate or high	3.86	(3.81-3.91)	3.79	(3.74-3.8 5)	2.8	(2.76-2.83)	2.69	(2.66-2.73)
		<0.001		<0.001		<0.001		0.02

model A—adjusted for age

model B—adjusted for age, height, BMI, smoking status, and respiratory disease.

SES transition was related with both FEV$_1$ and FVC. In men and women, participants whose SES decreased to low had pulmonary function as similar as participants who always had low SES. Compared to the groups of low SES or decreased SES to low, persons whose SES increased to moderate or high or persons whose SES was always on this level had higher FVC and FEV$_1$, although the differences in women were smaller compared to men (**tables 3–4**).

For peer review only

Discussion

The study showed that childhood and adulthood SES as well as life course changes in SES were associated with pulmonary function measured by FEV_1 and FVC. The relationship was attenuated slightly, particularly in men after adjustment for age, height, smoking, obesity, and history of respiratory diseases but remain statistically significant. The attenuation was mostly explained by height, which itself serves as a marker of early-life disadvantage and directly affects the lung function[28-30].

In this study, we applied a novel approach to identify uniquely relevant dimensions of childhood SES and adulthood SES from a range of socioeconomic indicators that we used. Additionally, we explored longitudinal SES exposure variable from childhood to adulthood, guided by two life-course SES hypotheses: 1) inter-generational social mobility, 2) cumulative disadvantage.

Results from our study, with reference to life stages of childhood or adulthood SES, are consistent with previous studies[21,29-33], which have shown that pulmonary function is gradually worse in the lower SES groups, although the magnitude of the effect varied across the studies.

Almost all previous studies used either single index of SES (education, income, or occupation) or attempted to explain the independent effect of one index, by adjusting for other indices in regression analysis. However, this approach is questioned as it does not take into account the joint effect of these factors and their interrelationship[17]. In our study, we used a model-based framework (cluster analysis) to identify SES groups that captured a detailed heterogeneity in education, previous and current economic situation, and current occupational status. As a result we observed that for the adult SES, low and middle SES did not differ in education and home amenities index. They differed significantly in their current occupational status, as low SES group was dominated by pensioners and unemployed people, which further, partially, explained the differences in current material resources for everyday living. The associations of the complex SES with pulmonary functions were in line with other studies[29-33].

Considering the childhood SES, parent's educational achievements overlapping slightly between low-middle as well as middle-high SES groups reflect the aspect that a single indicator of education does not sufficiently capture the complex pattern of SES characteristics. However,

For peer review only - http://bmjopen.bmj.com/site/about/guidelines.xhtml

the standard of housing (the second component of SES) separated the low and high groups unequivocally. In contrast to other studies, we did not find significant low–high SES differences in pulmonary measurements in men, after adjustments for adult risk factors. This suggests that the SES effect in childhood might be masked by SES in adulthood. Though parent's education significantly determines children's educational opportunity, during the socialist times the common national policy was to equal the chances for education by giving favors to children from lower socioeconomic groups. This policy reduced the inequality in education; however, because of quick industrialization of the country, education was strongly oriented to vocational level. Hazardous work and the prevalence of more frequent smoking in men may partially explain the lack of this significance[34-35].

The present study verified two hypotheses of several competing life-course conceptual models—social transition and cumulative SES frameworks. We showed that SES transition since childhood to adulthood was related with a functional lung state in adult life. To our knowledge, only one study has attempted to analyze SES trajectories during the life course in relation to lung function disparities. Menezes et al. have showed that SES at birth, in adolescence, and its trajectory between these two periods were inversely associated with lung function measurement by $FEV_1$and FVC in Brazilians[36].

Within the cumulative SES score, our results showed that the accumulation of social disadvantage is linked to poorer lung function in women; however, we did not find this pattern in men. After adjustment for adult SES-related covariates, graded associations with pulmonary measurements were attenuated, which suggests that childhood conditions are less important in men and it supports the significance of contemporary SES-related factors.

The mechanism of the association between SES and lung function is not fully understood. The biological pathway by which a limited lung development in prenatal period is related to infant respiratory infection and higher sensitivity to impaired lung function in adult is relatively well established. Another explanation is the sociobiological pathway: SES in childhood is adversely associated with postnatal lung function, which causes worse lung development as well as impact on the immune system through increased exposure to risk factors like air pollution, passive smoking, or poor nutrition. Respiratory diseases in childhood may be an obstacle in gaining the right education, which leads to lower SES in adulthood.

Differences in the respiratory system functioning among adults with certain SES may be explained by differences in the occupation—especially the intensity and frequency of physically hazardous work that has a direct impact on the respiratory system[34]. In addition, low SES is

For peer review only - http://bmjopen.bmj.com/site/about/guidelines.xhtml

associated with more frequent exposure to risk factors, such as nutrition disorders, smoking, more frequent infections, and obstructive pulmonary disease[34,36-39].

All the above factors are simultaneously related with the SES. That is why the most possible explanation for the relation between SES and the deterioration of respiratory functions is multifactorial due to simultaneous influence of many factors, which enables to explain, partially at least, the described relations.

Our study introduced some new aspects to current knowledge. Firstly, this study is the first in (CEE), which considers SES in the light of social and political transformations after the fall of the communist system. Secondly, we have defined model-based measures of childhood SES and adulthood SES that capture the multidimensionality of SES, which provide a detailed picture of the defined SES levels. This novel approach might facilitate the understanding of complexity of SES construct. For example, while about 60% of men from high SES childhood group had attained the university education, which would place them in higher SES group taking education as a single SES index, this percentage dropped to 47% for those belonging to high SES group assessed as composite measure. This marked the inconsistency in SES groups, which can be explained in two ways. First, levels of education do not go together with other dimensions of SES. Second, current economic situation (one of the elements of our SES composite measure) that provides resources to control one's circumstances overcomes the educational level among people who are nearly 60 years old and live in this part of Europe.

However, there are some limitations in the interpretation of the results. First, the cross-sectional study design does not allow address the causality of the observed relations. SES and, especially, its economical components are sensitive to changes over time. This may influence the health and then support health selection hypothesis, which in turn would strengthen the relation between SES and the studied health outcome. Secondly, SES in childhood (at 10 years) was assessed by interviews. Retrospective collection of information increases the random error and it is subject to recall bias. Furthermore, the known effect of the overestimation of socioeconomic position in childhood, obtained on the basis about father's/mother's education, might also bias the results.[40]

For peer review only - http://bmjopen.bmj.com/site/about/guidelines.xhtml

Conclusion

Low SES over life-course was associated with the lowest lung function. Downward social mobility was associated with poorer pulmonary function while upward mobility or life-course moderate or high SES were associated with better pulmonary function.

For peer review only

For peer review only - http://bmjopen.bmj.com/site/about/guidelines.xhtml

Funding:

The author(s) has disclosed the receipt of the following financial support for the research, authorship, and/or publication of this article: this work was funded by the Welcome Trust (grant WT064947 and WT081081), the US National Institute of Aging (grant R01 AG23522), and the MacArthur Foundation.

Contribution:

Maciej Polak drafted the manuscript, designed the analysis and did data analysis

Krystyna Szafraniec contributed to the data analysis and helped draft manuscript, critical revised the manuscript.

Magdalena Kozela was responsible for critical revision of the manuscript.

Renata Wolfhaut-Wolak was responsible for tables preparations and critical revision of the manuscript.

Martin Bobak contributed to the study design, critical revision of the manuscript.

Andrzej Pająk contributed to the study design, critical revision of the manuscript.

All authors approved the final version of the manuscript and this submission

Data sharing statement:

Data not in public domain

Competing Interest Statement:

All authors reports grants from The Welcome Trust, grants from The US National Institute of Aging, grants from The MacArthur Foundation during the conduct of the study. AP: personal fees from AMGEN, personal fees from Sanofi, outside the submitted work.

For peer review only - http://bmjopen.bmj.com/site/about/guidelines.xhtml

For peer review only

For peer review only - http://bmjopen.bmj.com/site/about/guidelines.xhtml

References

1 Kunst A. Socioeconomic inequalities in health in Central and Eastern Europe: synthesis of results of eight new studies. *Int J Public Health* 2009;54:197–200.

2 Mackenbach JP, Stirbu I, Roskam AJR, et al. Socioeconomic inequalities in health in 22 European countries. *New Engl J Med* 2008;358:2468–81.

3 Mackenbach JP, Huisman M, Andersen O, et al. Inequalities in lung cancer mortality by the educational level in 10 European populations. *Eur J Cancer* 2004;40:126–35.

4 Adler NE, Boyce T, Chesney MA, et al. Socioeconomic status and health: the challenge of the gradient. *Am. Psychol.* 1993 49:15–24.

5 Braveman PA, Cubbin C, Egerter S, et al. Socioeconomic disparities in health in the United States: What the patterns tell us. *Am J Public Health*, 2010. 100 (Suppl 1), 186–96

6 Stringhini S, Batty GD, Bovet , et al. Association of Lifecourse Socioeconomic Status with Chronic Inflammation and Type 2 Diabetes Risk: The Whitehall II Prospective Cohort Study. *PLoS Med* 2013 10(7): Published online 2013 Jul 2. e1001479. doi:10.1371/journal.pmed.1001479

7 Goodman E, Adler NE, Kawachi I, et al. Adolescents' perceptions of social status: development and evaluation of a new indicator. *Pediatrics* 2001. 108(2): E31.

8 Demakakos P, Nazroo J, Breeze E, et.al. Socioeconomic status and health: the role of subjective social Status. *Soc Sci Med*. 2008; 67(2): 330–340.

9 Ben-Shlomo Y, Kuh D. A life course approach to chronic disease epidemiology: conceptual models, empirical challenges and interdisciplinary perspectives. *Int J Epidemiol* 2002. 31(2): 285-93.

10 McEwen BS, Gianaros PJ. Central role of the brain in stress and adaptation: Links to socioeconomic status, health, and disease, *Ann N Y Acad Sci*. 2010; 1186: 190–222.

11 Richard PS, Huang M-H, Sidney S, et. al. Socioeconomic status and health: is parasympathetic nervous system activity an intervening mechanism? *Int J Epid* 2005;34:309–315

12 Beckie TM. A Systematic Review of Allostatic Load, Health, and Health Disparities, *Biological Research for Nursing* 2012, 14(4) 311-346

13 Fox AJ, Goldblatt PO, Jones DR. Social class mortality differentials: artefact, selection, or life circumstance. *J. Epidemiol. Commun. Health* 1985 39: 1–8.

14 Mulatu MS, Schooler C. Causal connections between socio-economic status and health: reciprocal effects and mediating mechanisms *J. Health Soc. Behav.* 2002, 43: 22-41

15 Mayer KU. New directions in life course research. *Ann Rev Sociology 2009*; 35: 413–433.

16 Laurie M, Corna A. life course perspective on socioeconomic inequalities in health: A critical review of conceptual frameworks, *Adv Life Course Res*. 2013,18: 150–159

17 Cutler DM, Lleras-Muney A, Vogl T. Socioeconomic Status and Health: Dimensions and Mechanisms. Publication Date: Sep 2012. DOI:10.1093/oxfordhb/9780199238828.013.0007

For peer review only - http://bmjopen.bmj.com/site/about/guidelines.xhtml

18 Marin TJ, Chen E, Miller GE. What Do Trajectories of Childhood Socioeconomic Status Tell Us About Markers of Cardiovascular Health in Adolescence? *Psychosom Med* 2008, 70:152–159

19 Matthews KA, Kiefe CI, Lewis CE, et al. Socioeconomic Trajectories and Incident Hypertension in a Biracial Cohort of Young Adults. *Hypertension* 2002;39:772-776.

20 Kozyrskyj AL, Kendall GE, Jacoby P, Sly PD, et al. Association Between Socioeconomic Status and the Development of Asthma: Analyses of Income Trajectories. *Am J Public Health*. 2010;100:540–546.

21 Tabak C, Spijkerman AMW, Verschuren WMM, et al. Does educational level influence lung function decline (Doetinchem Cohort Study)? *Eur Respir J* 2009; 34: 940–947

22 Schünemann HJ, Dorn J, Grant BJB, et al. Pulmonary Function Is a Long-term Predictor of Mortality in the General Population*: 29-Year Follow-up of the Buffalo Health Study *Chest* 2000;118(3):656-664.

23 Stavem K, Aaser E, Sandvik L, et al. Lung function, smoking and mortality in a 26-year follow-up of healthy middle-aged Males. *Eur Respir J* 2005; 25: 618–625

24 Johannessen A, Eagan TML, Omenaas ER, et al. Socioeconomic risk factors for lung function decline in a general population A. *Eur Respir J* 2010; 36: 480–487

25 Peasey A, Bobak M, Kubinova R, et al. Determinants of cardiovascular disease and other non-communicable disease in Central and Eastern Europe: rationale and design of the HAPIEE study. BMC Public Health. 2006;6:255.

26 Miller MR, Hankinsosn J, Brusasco V, et al. Standardisation of spirometry. *Eur. Respir. J. 2005* 26, 319-338.

27 Ciu T, Fang D, Chen J, and Jerois , C. (2001). A Robust and Scalable Clustering Algorithm, for Mixed Type Attributes in Large Database Environment. *In: Proceedings of the seventh ACM SIGKDD international conference on knowledge discovery and data mining. San Francisco, CA: ACM 2001.*

28 Gray LA, Leyland AH, Benzeval M, et al. Explaining the social patterning of lung function in adulthood at different ages: the roles of childhood precursors, health behaviours and environmental factors. *J Epidemiol Community Health*. 2013;67(11):905–11.

29 McFadden E, Luben R, Wareham N, et al. How far can we explain the social class differential in respiratory function? A cross-sectional population study of 21,991 men and women from EPIC-Norfolk. *Eur J Epidemiol*. 2009;24(4):193–201

30 Merkus PJ, ten Have-Opbroek AA, Quanjer PH Human lung growth: areview. *Pediatr Pulmonol* 1996, 21(6):383-397.

31 Hegewald MJ, Crapo RO. Socioeconomic status and lung function. *Chest*. 2007;132(5):1608–14.

32 Gaffney AW, Hang J, Lee MS, et al. Socioeconomic status is associated with reduce lung function in China: an analysis from large cross-sectional study in Shanghai. *BMC Public Health* 2016;16:96

33 Ramsay SE, Whincup PH, Lennon LT, et al. Longitudinal associations of socioeconomic position in childhood and adulthood with decline in lung function over 20 years: results from a population-based cohort of British men. *Thorax*. 2011 Dec;66(12):1058-64.

34 Krzyzanowski M, Jedrychowski W. Occupational exposure and incidence of chronic respiratory symptoms among residents of Cracow followed for 13 years. Int Arch Occup Environ Health. 1990;62(4):311-7

35 Przewozniak K. , Sulkowska U, et al.. Tobacco smoking in countries of the European Union. Ann Agric Environ Med. 2012;19(2):181-92.

For peer review only - http://bmjopen.bmj.com/site/about/guidelines.xhtml

36 Menezes MB, Dumith SC, Perez-Padilla R, et al. Socioeconomic trajectory from birth to adolescence and lung function: prospective birth cohort study. *BMC Public Health*, 2011, 11:596

37 Shaheen SO, Jameson KA, Syddall HE, et al. The relationship of dietary patterns with adult lung function and COPD. *Eur Respir J*. 2010;36(2):277–84.

38 Tabak C, Smit HA, Rasanen L, et al. Dietary factors and pulmonary function: a cross sectional study in middle aged men from three European countries. *Thorax*. 1999;54(11):1021–6.

39 Strachan DP. Ventilatory function, height, and mortality among lifelong non-smokers. *J Epidemiol Community Health* 1992; 46: 66–70.

40 Batty GD, Lawlor DA, Macintyre S, et al. Accuracy of adults' recall of childhood social class: findings from the Aberdeen children of the 1950s study. *J Epidemiol Community Health* 2005;59:898-903.

For peer review only

For peer review only - http://bmjopen.bmj.com/site/about/guidelines.xhtml

STROBE Statement—checklist of items that should be included in reports of observational studies

	Item No	Recommendation
**Title and abstract**	1	*(a)* Indicate the study's design with a commonly used term in the title or the abstract – page 1
		(*b*) Provide in the abstract an informative and balanced summary of what was done and what was found – page 2
**Introduction**		
Background/rationale	2	Explain the scientific background and rationale for the investigation being reported – page 4
Objectives	3	State specific objectives, including any prespecified hypotheses page 4
**Methods**		
Study design	4	Present key elements of study design early in the paper – page 5
Setting	5	Describe the setting, locations, and relevant dates, including periods of recruitment, exposure, follow-up, and data collection – page 5
Participants	6	(*a*) *Cohort study*—Give the eligibility criteria, and the sources and methods of selection of participants. Describe methods of follow-up<br>*Case-control study*—Give the eligibility criteria, and the sources and methods of case ascertainment and control selection. Give the rationale for the choice of cases and controls<br>*Cross-sectional study*—Give the eligibility criteria, and the sources and methods of selection of participants – page 5
		(*b*) *Cohort study*—For matched studies, give matching criteria and number of exposed and unexposed<br>*Case-control study*—For matched studies, give matching criteria and the number of controls per case
Variables	7	Clearly define all outcomes, exposures, predictors, potential confounders, and effect modifiers. Give diagnostic criteria, if applicable – page 5-7
Data sources/ measurement	8*	For each variable of interest, give sources of data and details of methods of assessment (measurement). Describe comparability of assessment methods if there is more than one group – page 5-6
Bias	9	Describe any efforts to address potential sources of bias page – page 15
Study size	10	Explain how the study size was arrived at - page 9
Quantitative variables	11	Explain how quantitative variables were handled in the analyses. If applicable, describe which groupings were chosen and why – page 8
Statistical methods	12	(*a*) Describe all statistical methods, including those used to control for confounding– page 8
		(*b*) Describe any methods used to examine subgroups and interactions– page 8
		(*c*) Explain how missing data were addressed– page 8
		(*d*) *Cohort study*—If applicable, explain how loss to follow-up was addressed<br>*Case-control study*—If applicable, explain how matching of cases and controls was addressed<br>*Cross-sectional study*—If applicable, describe analytical methods taking account of sampling strategy
		(*e*) Describe any sensitivity analyses

For peer review only

**Results**		
Participants	13*	(a) Report numbers of individuals at each stage of study—eg numbers potentially eligible, examined for eligibility, confirmed eligible, included in the study, completing follow-up, and analysed – page 9
		(b) Give reasons for non-participation at each stage
		(c) Consider use of a flow diagram
Descriptive data	14*	(a) Give characteristics of study participants (eg demographic, clinical, social) and information on exposures and potential confounders – page 9-10
		(b) Indicate number of participants with missing data for each variable of interest – page 9
		(c) *Cohort study*—Summarise follow-up time (eg, average and total amount)
Outcome data	15*	*Cohort study*—Report numbers of outcome events or summary measures over time
		Case-control study—Report numbers in each exposure category, or summary measures of exposure
		Cross-sectional study—Report numbers of outcome events or summary measures – pages9-11
Main results	16	(*a*) Give unadjusted estimates and, if applicable, confounder-adjusted estimates and their precision (eg, 95% confidence interval). Make clear which confounders were adjusted for and why they were included – page 10-12
		(*b*) Report category boundaries when continuous variables were categorized – not applicable
		(*c*) If relevant, consider translating estimates of relative risk into absolute risk for a meaningful time period
Other analyses	17	Report other analyses done—eg analyses of subgroups and interactions, and sensitivity analyses – page 9-10
**Discussion**		
Key results	18	Summarise key results with reference to study objectives – page 13
Limitations	19	Discuss limitations of the study, taking into account sources of potential bias or imprecision. Discuss both direction and magnitude of any potential bias – page 15
Interpretation	20	Give a cautious overall interpretation of results considering objectives, limitations, multiplicity of analyses, results from similar studies, and other relevant evidence – page 14-15
Generalisability	21	Discuss the generalisability (external validity) of the study results– page 13-14
**Other information**		
Funding	22	Give the source of funding and the role of the funders for the present study and, if applicable, for the original study on which the present article is based– page 17

*Give information separately for cases and controls in case-control studies and, if applicable, for exposed and unexposed groups in cohort and cross-sectional studies.

Note: An Explanation and Elaboration article discusses each checklist item and gives methodological background and published examples of transparent reporting. The STROBE checklist is best used in conjunction with this article (freely available on the Web sites of PLoS Medicine at http://www.plosmedicine.org/, Annals of Internal Medicine at http://www.annals.org/, and Epidemiology at http://www.epidem.com/). Information on the STROBE Initiative is available at www.strobe-statement.org.

For peer review only

BMJ Open

Socioeconomic status and pulmonary function, transition from childhood to adulthood: cross-sectional results from the Polish part of the HAPIEE study.

Journal:	*BMJ Open*
Manuscript ID	bmjopen-2018-022638.R1
Article Type:	Research
Date Submitted by the Author:	19-Jun-2018
Complete List of Authors:	Polak, Maciej; Jagiellonian University Medical College, Krakow, Poland, Chair of Epidemiology and Population Studies, Faculty of Health Sciences,<br>Szafraniec, Krystyna; Jagiellonian University Medical College, Krakow, Poland, Chair of Epidemiology and Population Studies, Faculty of Health Sciences<br>Kozela, Magdalena ; Jagiellonian University Medical College, Krakow, Poland, Chair of Epidemiology and Population Studies, Faculty of Health Sciences,<br>Wolfshaut-Wolak, Renata; Jagiellonian University Medical College, Krakow, Poland, Chair of Epidemiology and Population Studies, Faculty of Health Sciences<br>Bobak, Martin; University College London, Epidemiology and Public Health<br>Pająk, Andrzej; Jagiellonian University Medical College, Krakow, Poland, Chair of Epidemiology and Population Studies, Faculty of Health Sciences
**Primary Subject Heading**:	Epidemiology
Secondary Subject Heading:	Sociology
Keywords:	SES, Lung function, Social mobility

SCHOLARONE™
Manuscripts

Title:

Socioeconomic status and pulmonary function, transition from childhood to adulthood: cross-sectional results from the Polish part of the HAPIEE study

Maciej Polak, Krystyna Szafraniec, Magdalena Kozela, Renata Wolfshaut-Wolak, Martin Bobak, Andrzej Pająk

Affiliation: Chair of Epidemiology and Population Studies, Institute of Public Health, Jagiellonian University Medical College

Corresponding author:

Maciej Polak

Chair of Epidemiology and Population Studies
Faculty of Health Science
Jagiellonian University Medical College
31-531 Kraków, ul. Grzegórzecka 20
phone: +48 12 43 32 837
e-mail: maciej.1.polak@uj.edu.pl

For peer review only - http://bmjopen.bmj.com/site/about/guidelines.xhtml

Abstract

Objective

Previous studies reported inverse associations between socioeconomic status (SES) and lung function but less is known whether pulmonary function is affected by SES changes.

We aimed to describe the relation of changes of SES between childhood and adulthood with pulmonary function.

Design

Cross-sectional study.

Participants

The study sample included 4104 men and women, aged 45–69 years, residents of Krakow, participating in the Polish part of the HAPIEE Project

Main outcome

Forced expiratory volume (FEV_1) and forced vital capacity (FVC) were assessed by the standardized spirometry procedure. Participants were classified into the 3 categories of SES (low, moderate, or high) based on the information on parent's education, housing standard during childhood, own education, professional activity, household amenities, and financial status.

Results

The adjusted difference in mean FVC between persons with low and high adulthood SES was 100 ml (p=0.005) in men and 100 ml (p<0.001) in women; the differences in mean FEV_1 were 103 ml (p<0.001) and 80 ml (p<0.001), respectively. Participants who changed SES during life course from low at childhood to moderate or high at adulthood and those who had moderate or high SES at both childhood and adulthood had significantly higher FEV_1 and FVC compared to those who had always low SES or SES changed to low.

Conclusions

Low SES over life course was associated with the lowest lung function. Downward social mobility was associated with poorer pulmonary function while upward mobility or life course moderate or high SES were associated with better pulmonary function.

For peer review only - http://bmjopen.bmj.com/site/about/guidelines.xhtml

Strengths and limitations of this study.

Strengths

- Addresses the association between social mobility and pulmonary function assessed by objectively measured FVC and FEV_1.
- This is the first study to confirm consistency of the described association between SES and pulmonary function in Eastern Europe, a region undergoing profound social and political transformation after the fall of the communism.
- Multidimensional nature of SES was captured by application of cluster analysis.

Limitation

- The cross-sectional study design does not allow address the causality of the observed relations.
- Information on childhood SES was based on a distant recall of various markers of social circumstances. This could have contributed to overestimation of childhood SES.

For peer review only

For peer review only - http://bmjopen.bmj.com/site/about/guidelines.xhtml

Introduction

Socioeconomic status (SES) is one of the main predictors of health, persisted at each stage of the life cycle[1-3]. People in lower socioeconomic group usually experience poorer health than people from higher socioeconomic group, regardless of the health measures and SES indicators used[4-6]. SES is a complex, multidimensional social construct conceptualized by combination of income, education, and occupation (and other factors) that are often interrelated. However, it is likely that each SES measure reflects slightly different forces associated with health and disease on individual and societal level[7].

Several social, psychological, and biological mechanisms have been proposed to explain the association of SES with health[8-10]. It has been advocated that SES cannot directly affect health as do physiological variables but that it acts indirectly through behavioural factors or chronic stress that determine health[8]. In addition, people of higher socioeconomic status are also more likely to utilize knowledge, financial resources and beneficial social networks in order to evaluate risk factors and take up protective actions. On the other hand, there is also some evidence for reverse hypothesis on health to SES relationship: "social drift" links poor health to a subsequent downward shift in the social class[11-12].

Studies that focus on determinants of adult disease frequently ignore the influence of factors coming from previous stages of life. Standard practice for measuring SES in most of health studies is to include measures of current socioeconomic characteristics. Even if childhood socioeconomic position is measured, it is usually accounted in multivariable models without taking into account the temporal relationship between variables[13]. The life course approach to how SES affects health has been increasingly applied to studying health[14-16]. The life course framework consists of several, partially interrelated models. The most frequently used concepts regarding the influence of life course SES on heath are: 1/ the critical period model emphasizing the importance of the timing of SES exposure, 2/ a social mobility model considers the influence of changes in marker of SES over time followed by upward mobility or downward mobility, which in turn may impact health, 3/ the cumulative exposure model which hypothesizes that factors acting at different stages of life accumulate to influence disease risk in later life[9,17]. Thus, the life course approach allows to go beyond simple measures of childhood and adulthood SES to better capture important aspects of SES exposure for disease development. Whilst the life course approach has already been implemented to study the effects of SES on CVD[18], obesity[5], hypertension[19], and diabetes[6], it has been applied less frequently to respiratory diseases[20,21].

For peer review only - http://bmjopen.bmj.com/site/about/guidelines.xhtml

Lung function is a long-term predictor of morbidity and mortality from a range of diseases including non-respiratory conditions[22,23]. Studies have demonstrated the association of a decrease in lung function not only with increasing age, but also with low SES[24]. However, little is known how the changes in SES patterns over the influence the respiratory health. None of the research from Central and Eastern Europe (CEE) examined this association and hence this topic becomes challenging. Especially because of political transformation of 1989/90 occurring in this region, followed by rapid social and economic changes, resulted in widening income inequalities and changes in health behavior. Additionally, most of middle-aged people's life crossed over that time period resulting in some phenomena that childhood SES was well established before and adulthood SES have been developing after the fall of socialism.

The aim of this study was to examine socioeconomic gradient in pulmonary function in middle age adults. The analysis was guided by three research questions, all aimed at characterizing socioeconomic patterning of lung functions after adjustment for modifiable risk factors such as smoking and body mass index (BMI): (1) whether there is a gradual (dose related) association between childhood and adulthood SES and pulmonary function measured by forced vital capacity (FVC) and one-second forced expiratory volume (FEV_1); (2) whether there is a relationship between social mobility and pulmonary function; and (3) whether there is a relation between cumulative SES and pulmonary function.

For peer review only - http://bmjopen.bmj.com/site/about/guidelines.xhtml

Methods

Study design and data collection

The analysis was performed using data from the Polish part of the HAPIEE (*Health, Alcohol and Psychosocial Factors In Eastern Europe*) study. The design of this study has been described in detail elsewhere[25]. In summary, the study included four urban cohorts selected from population registers in Novosibirsk (Russia), Krakow (Poland), six Czech towns and Kaunas (Lithuania); each consisted of a random sample of men and women aged 45–69 years old at baseline in 2002-2005. All participants completed a comprehensive health questionnaire and underwent a brief medical examination including pulmonary function test. In Poland trained nurses interviewed the respondents at their homes. For this analysis information about age, current and past socioeconomic circumstances, health status (asthma or history of respiratory disease), and health behavior, i.e., smoking and physical activity was obtained using a standard questionnaire. Then all participants were invited to a clinic for anthropometric and blood pressure measurements followed by blood collection for biochemical tests and for performing spirometry.

Patient and Public Involvement statement

Participants were not involved in the design of this study.

Measures

Pulmonary function assessment

In our analysis we used two parameters of pulmonary function: FVC, which is recognized as a predictor of vital status, and FEV_1, which is regarded as a good biological marker of risk of obstructive pulmonary disease. FVC and FEV_1 were measured by a trained technician in accordance with a standardized protocol[26] using Micro-Medical Microplus spirometer. Participants were asked to make at least three maximal expiratory measures. If the quality of the spirometry was not satisfactory, the maneuver was repeated until the best quality was obtained. The highest value of FVC and the highest value of FEV_1 were chosen from these measurements for which the repeatability criteria were met. The quality of all tests was reviewed by a pulmonologist. Criteria for a satisfactory repeatability were based on the European Respiratory Society recommendations[26]. Exclusion criteria for the spirometry

For peer review only - http://bmjopen.bmj.com/site/about/guidelines.xhtml

included any chest surgery during last three months, any physician-diagnosed serious cardiac condition (stroke, myocardial infarction), and recent respiratory infections.

Socioeconomic Status

Childhood SES was assessed using the level of education of both parents (incomplete primary or no formal education, primary, vocational, secondary, or university) and information about housing standard at the age of about 10 (cold tap water, hot tap water, radio, fridge, own kitchen, and own toilet), all summed up into an index measuring the amenities in childhood.

Adulthood SES was measured by five items: the study of participant's educational attainment, professional activity, household amenities, and current financial situation. Participant's education was assessed by questioning his/her highest level of education completed (incomplete primary or no formal education, primary, vocational, secondary, or university). Current employment status encompassed categories: entrepreneur, employed and self-employed, unemployed including housewife, and retired people. Index of household amenities was constructed by summing up the number of valuable items that a participant had in his household (microwave, washing machine, video recorder, colour television, dishwasher, freezer, camcorder, satellite TV, telephone and mobile phone). Current financial status was assessed by two questions: "How often does it happen not to have enough money for food which you and your family need?" and "Do you have any difficulties with paying bills?". The possible answers were the following: all the time, often, sometimes, rarely, or never. The same indicators were used in other reports from the HAPIEE study[27].

Two-step clustering algorithm was applied to classify participants into homogeneous groups of childhood SES and adulthood SES. In our analysis we allowed the process to identify the dimensions of SES without constraint to any particular number of factors, in order to capture the natural complexity of SES. Three groups of childhood SES were generated, assessing reasonable evidence of the cluster structure (silhouette coefficient s(i)=0.51). Participants who had poor housing conditions (low value of childhood amenities index) and their parents had no formal education or had at the most primary school were classified by the algorithm as a low SES group. The middle SES group comprised of subjects characterized by medium housing condition in childhood and secondary education completed by at least one parent of the participants. High value of childhood amenities index and parent's secondary or

For peer review only - http://bmjopen.bmj.com/site/about/guidelines.xhtml

university education classified a participant into the high childhood SES group. Similarly, three groups of adult SES were identified (s(i)=0.55). Participants who had a university education, were employed, and had a high index of amenities were classified by the cluster algorithm as having high SES in adulthood. On the other hand, participants with low educational achievements, unemployed, being on retirement pension, or reporting lack of money for food or paying bills have been classified into low SES group. The middle SES group comprised of participants characterized mainly by secondary education (however, 44% had a primary of education), half-and-half working and retired persons having no problem with money for food and paying bills regularly (**table 1**).

Table 1. Definition of the socioeconomic status

Socioeconomic status		Descriptive statistics	Cluster description
Childhood	Low	n=1632<br>Age (years): 58.9<br>Female (%):51	- Primary parent's education<br>- Low value of amenities index in childhood
	Middle	n=1463<br>Age (years): 58.4<br>Female (%): 51	- Primary or secondary parent's education<br>- Low or middle value of amenities index in childhood
	High	n=881<br>Age (years): 56.0<br>Female (%): 50	- Secondary or university parent's education<br>- High value of amenities index in childhood
Adulthood	Low	n=1313<br>Age (years): 57.8<br>Female (%): 58	- Primary or secondary education<br>- Often or sometimes do not have enough money for food or have problems with paying bills<br>- Unemployed or pensioner
	Middle	n=1588<br>Age (years): 58.9<br>Female (%): 49	- Primary or secondary education<br>- Rarely or never don't have enough money for food or have problems with paying bills
	High	n=1101<br>Age (years): 57.1<br>Female (%): 44	- University education<br>- Employed or owner of company<br>- High value of amenities index

Cumulative SES score was calculated by summing the childhood and adulthood SES evaluations obtained by cluster analysis with code 0 for low SES, code 1 for medium SES and code 2 for high SES. The final cumulative SES score was further categorized as low (score=0–1), medium (score=2–3), and high (score=4).

For peer review only - http://bmjopen.bmj.com/site/about/guidelines.xhtml

For peer review only

Transition from childhood to adulthood (social mobility)

Having separate estimates for early childhood SES and adult SES, it allowed each participant to be allocated to one of the following four separate group of SES changes: participants who were classified in the low subgroups in both time points were categorized as “always low”. Those who were classified in the low SES in childhood, but in adulthood in middle or high were categorized as “upward mobility”. Similarly, those classified in middle or high SES in childhood and low in adulthood were categorized as “downward mobility”. The categories moderate and high SES were combined due to similar differences in pulmonary function of participants who changed SES from low to moderate and from low to high SES. Finally, those classified in both life stages in the middle or high SES were categorized as “always moderate or high”.

Statistical analysis

Descriptive statistics stratified by sex were presented as means and standard deviations for pulmonary function parameters and other continuous measurements and as frequency (n, %) for categorical variables. Two-step cluster analysis was used to determine homogenous subgroups of childhood SES and adult SES. The procedure is particularly designed to handle large data set and allows considering both continuous and categorical variables. In the first step the algorithm uses a sequential clustering method to allocate the cases into many small sub-clusters. The second step takes sub-clusters resulting from the first step as input in order to group them into clusters. In the modeling process, the hierarchical clustering method and the log-likelihood distance measure between clusters were used. The validation of consistency within clusters of data was based on silhouette coefficient s(i). Values above 0.5 are recognized as either reasonable or strong evidence of cluster structure (details has been briefly described in[28]).

To compare FVC and FEV_1, the distribution by SES in childhood, in adulthood, and in transition of SES between these two periods, a set of analysis of covariance were performed separately for men and women. Due to ordinal character of the SES variables, p-values for linear trends in mean values of spirometry parameters (FEV_1 and FVC, separately) are presented. The final models were adjusted for age, height, BMI, smoking, and prevalence of respiratory diseases. Data was analyzed using IBM® SPSS® ver.22. All tests were two-tailed, p-value less than 0.05 was considered statistically significant.

For peer review only - http://bmjopen.bmj.com/site/about/guidelines.xhtml

For peer review only

Results

Table 2. Characteristics of the studied sample by sex

	Male n=2020		Female n=2084		p
	mean	SD	mean	SD	
Age	57.9	6.86	57.2	6.962	0.001[A]
BMI	28.10	4.130	28.47	5.040	0.016[A]
FEV_1 [L]	3.15	0.659	2.27	0.473	<0.001[A]
FVC [L]	3.81	0.717	2.73	5.280	<0.001[A]
	n	%	n	%	
Respiratory diseases (yes)	266	13.4	285	13.8	0.6[B]
Smoking status					
Current smoker	666	33.1	539	25.9	
Ex-smoker	755	37.5	419	20.2	<0.001[B]
Never smoker	592	29.4	1121	53.9	
Adulthood SES					
Low	548	27.1	765	36.7	
Middle	804	39.8	784	37.6	<0.001[B]
High	614	30.4	487	23.9	
Childhood SES					
Low	799	40.7	833	41.3	
Middle	723	36.9	740	36.7	0.9[B]
High	439	22.4	442	21.9	
Social mobility					
Always low	225	11.7	314	15.9	
Downward mobility	308	16.1	424	21.5	<0.001[B]
Upward mobility	557	29.1	503	25.5	
Always moderate or high	825	43.1	732	37.1	
Cumulative SES score					
Low	833	43.5	996	50.5	
Middle	729	43.3	769	39	<0.001[B]
High	253	13.2	208	10.5	

A–p value for t-test
B–p value for chi-square test

Out of 4840 participants for whom spirometry measurements were performed, 4104 (84.8%) participants provided valid spirometry data and were included in this analysis. There was no difference in age, BMI, smoking, and SES level in childhood between analyzed group and excluded participants. But, the latter group had a higher prevalence of respiratory diseases and higher percentage (by 7%) of low SES category in adulthood. The characteristics of the study sample are presented in **table 2**. The mean age was 55.6 (SD=6.92) years; men were slightly

For peer review only - http://bmjopen.bmj.com/site/about/guidelines.xhtml

older than women (57.9 years vs. 57.2 years). Mean FEV_1 for the whole sample was 2.70 L (95% CI 2.68 to 2.73), men had higher mean FEV_1 by 0.88 L, compared to women. Mean FVC was 3.25 L (95% CI 3.22 to 3.28), difference between men and women in mean FVC was statistically significant and was equal to 1.08 L. Distribution of SES indicators (adulthood, cumulative, and transition) differed between sexes. Low SES in adult was observed in 27% of men and 37% of women ($p<0.001$). No differences in the distribution of childhood SES emerged from sex groups.

Tables 3 and **4** show means of FEV_1 and FVC, related to SES indices (childhood SES, adulthood SES, cumulative SES and social mobility), in model adjusted for age and model adjusted to all other covariates. Age adjusted means of FEV_1 and FVC were associated with childhood (in women), adulthood, and cumulative SES score with significant gradient in men and women. Compared to high SES, men in the low or middle SES had significantly worse lung function (differences ranging from 0.20 L to 0.27 L in FEV_1 and 0.10 L to 0.26 L in FVC). Results were similar but less pronounced in women (differences from 0.07 L to 0.16 L in case of FEV_1 and 0.18 L to 0.25 L in FVC). Controlling for age, height, BMI, smoking, and prevalence of respiratory diseases resulted in some attenuation of the estimates, but the pattern and significance of the association remained, except for the relation between childhood SES and FVC, as well as cumulative SES and FEV_1 and FVC in men.

For peer review only

For peer review only - http://bmjopen.bmj.com/site/about/guidelines.xhtml

Table 3. **Means of FEV_1 [L] by category of childhood, adulthood SES, transition SES, and cumulative assessment of SES.**

	Male				Female			
	model A		model B		model A		model B	
Adulthood SES	mean	95% CI	mean	95% CI	men	95% CI	mean	95% CI
Low	3.01	(2.96-3.06)	2.9	(2.85-2.94)	2.22	(2.19-2.25)	2.12	(2.09-2.15)
Middle	3.16	(3.12-3.20)	2.99	(2.95-3.03)	2.27	(2.24-2.30)	2.15	(2.12-2.18)
High	3.28	(3.24-3.32)	3.03	(2.98-3.07)	2.36	(2.33-2.40)	2.2	(2.16-2.24)
p for trend	<0.001		<0.001		<0.001		0.02	
Childhood SES	mean	95% CI	mean	95% CI	mean	95% CI	mean	95% CI
Low	3.15	(3.11-3.19)	3.00	(2.95-3.04)	2.27	(2.24-2.29)	2.16	(2.13-2.19)
Middle	3.18	(3.08-3.27)	2.95	(2.90-2.99)	2.25	(2.22-2.28)	2.12	(2.09-2.16)
High	3.19	(3.14-3.25)	2.94	(2.89-3.00)	2.34	(2.30-2.37)	2.19	(2.15-2.23)
p for trend	0.1		0.1		0.001		0.011	
Cumulative SES score	mean	95% CI	mean	95% CI	mean	95% CI	mean	95% CI
Low	3.09	(3.05-3.13)	2.95	(2.91-3.00)	2.24	(2.18-2.24)	2.14	(2.11-2.17)
Middle	3.18	(3.14-3.22)	2.97	(2.93-3.01)	2.29	(2.26-2.32)	2.15	(2.12-2.18)
High	3.29	(3.22-3.36)	2.99	(2.92-3.06)	2.4	(2.35-2.46)	2.23	(2.17-2.28)
p for trend	<0.001		0.6		<0.001		0.009	
Social mobility		95% CI	mean	95% CI	mean	95% CI	mean	95% CI
Always low	2.98	(2.90-3.05)	2.91	(2.84-2.98)	2.21	(2.17-2.26)	2.14	(2.10-2.19)
Downward mobility	3.03	(2.97-3.10)	2.88	(2.82-2.94)	2.23	(2.19-2.27)	2.13	(2.09-2.17)
Upward mobility	3.21	(3.16-3.26)	3.03	(2.98-3.08)	2.3	(2.27-2.34)	2.19	(2.15-2.23)
Always moderate or high	3.21	(3.17-3.24)	3.04	(2.98-3.07)	2.31	(2.28-2.34)	2.21	(2.18-2.24)
p for trend	<0.001		<0.001		<0.001		0.048	

model A—adjusted for age

model B—adjusted for age, height, BMI, smoking status and respiratory disease.

For peer review only

Table 4. **Means of FVC [L] in category of childhood, adulthood, and transition SES and cumulative assessment of SES.**

	Male				Female			
	model A		model B		model A		model B	
Adulthood SES	mean	95% CI	mean	95% CI	mean	95% CI	mean	95% CI
Low	3.66	(3.61-3.72)	3.61	(3.55-3.67)	2.67	(2.64-2.71)	2.63	(2.59-3.66)
Middle	3.83	(3.78-3.88)	3.73	(3.68-3.78)	2.71	(2.67-2.74)	2.65	(2.61-2.69)
High	3.92	(3.86-3.97)	3.71	(3.65-3.76)	2.85	(2.81-2.89)	2.73	(2.68-2.77)
p for trend	<0.001		0.005			<0.001		<0.001
Childhood SES	mean	95% CI	mean	95% CI	mean	95% CI	mean	95% CI
Low	3.79	(3.74-3.83)	3.71	(3.66-3.76)	2,69	(2.66-2.72)	2.642	(2.61-2.68)
Middle	3.77	(3.72-3.82)	3.66	(3.60-3.71)	2.71	(2.68-2.75)	2.635	(2.60-2.67)
High	3.89	(3.83-3.96)	3.69	(3.62-3.75)	2.84	(2.79-2.88)	2.725	(2.68-2.77)
p for trend		0.001		0.1		<0.001		0.001
Cumulative SES score	mean	95% CI	mean	95% CI	mean	95% CI	mean	95% CI
Low	3.73	(3.67-3.78)	3.67	(3.62-3.72)	2.67	(2.64-2.70)	2.63	(2.60-2.67)
Middle	3.84	(3.80-3.89)	3.69	(3.64-3.74)	2.76	(2.72-2.79)	2.66	(2.62-2.70)
High	3.93	(3.84-4.02)	3.67	(3.58-3.75)	2.92	(2.85-2.98)	2.78	(2.71-2.84)
p for trend		<0.001		0.7		<0.001		<0.01
Social mobility		95% CI	mean	95% CI	mean	95% CI	mean	95% CI
Always low	3.59	(3.50-3.68)	3.6	(3.52-3.67)	2.64	(2.58-2.69)	2.62	(2.57-2.67)
Downward mobility	3.72	(3.64-3.80)	3.63	(3.56-3.70)	2.71	(2.66-2.75)	2.64	(2.59-2.68)
Upward mobility	3.87	(3.81-3.92)	3.77	(3.71-3.83)	2.72	(2.68-2.76)	2.67	(2.63-2.72)
Always moderate or high	3.86	(3.81-3.91)	3.79	(3.74-3.8 5)	2.8	(2.76-2.83)	2.69	(2.66-2.73)
p for trend		<0.001		<0.001		<0.001		0.02

model A—adjusted for age

model B—adjusted for age, height, BMI, smoking status, and respiratory disease.

For peer review only

For peer review only - http://bmjopen.bmj.com/site/about/guidelines.xhtml

Social mobility was related to both FEV_1 and FVC. In men and women, participants whose SES decreased to low had pulmonary function similar to participants who always had low SES. Compared to persons with life course low SES or SES decreased to low, persons with life course moderate or high SES or increased SES, had higher FVC and FEV_1. The differences between participants who always had low SES compared to those with moderate or high SES were 190 ml for FVC and 130 ml for FEV_1, in men. In women the differences were smaller and equal to 70 ml for both FVC and FEV_1 (**tables 3–4**).

For peer review only

Discussion

In this study, we applied a novel approach to identify uniquely relevant dimensions of childhood SES and adulthood SES from a range of socioeconomic indicators that we used . The study showed that childhood and adulthood SES as well as life course changes in SES were associated with pulmonary function measured by FEV_1 and FVC. The relationship was attenuated slightly, particularly in men after adjustment for age, height, smoking, obesity, and history of respiratory diseases but remained statistically significant. The attenuation was mostly explained by height, which itself serves as a marker of early-life disadvantage and directly affects the lung function[29-31].

Results from our study, with reference to life stages of childhood or adulthood SES, are consistent with previous studies[21,30-34], which have shown that pulmonary function is gradually worse in the lower SES groups, although the magnitude of the effect varied across the studies.

Almost all previous studies used either indicator of SES (education, income, or occupation) or attempted to explain the independent effect of one indicator, by adjusting for other indices in regression analysis. However, this approach is questioned as it does not take into account the joint effect of these factors and their interrelationship[16]. In our study, we used a model-based framework (cluster analysis) to identify SES groups that captured a detailed heterogeneity in education, previous and current economic situation, and current occupational status. Cluster analysis is not a traditional approach to social stratification, although it was applied in some studies[35-36]. This methods seems to be particularly suitable for CEE society in which social classes are less clearly separated from each other. As a result we observed that for the adult SES, low and middle SES did not differ in education and home amenities index. They differed significantly in their current occupational status, as low SES group was dominated by pensioners and unemployed people, which further, partially, explained the differences in current material resources for everyday living. The associations of the complex SES with pulmonary functions were in line with other studies[30-34].

Considering the childhood SES, parent's educational achievements overlapping slightly between low-middle as well as middle-high SES groups reflect the aspect that a single indicator of education does not sufficiently capture the complex pattern of SES characteristics. However, the standard of housing (the second component of SES) separated the low and high groups unequivocally. In contrast to other studies, we did not find significant low–high SES differences

For peer review only - http://bmjopen.bmj.com/site/about/guidelines.xhtml

in pulmonary measurements in men, after adjustments for adult risk factors. This suggests that the SES effect in childhood might be masked by SES in adulthood. Though parent's education significantly determines children's educational opportunity, during the socialist times the common national policy was to equal the chances for education by giving favors to children from lower socioeconomic groups. This policy reduced the inequality in education; however, because of quick industrialization of the country, education was strongly oriented to vocational level. Hazardous work and the prevalence of more frequent smoking in men may partially explain the lack of this significance[37-38].

The present study verified two hypotheses of several competing life course conceptual models - social mobility and cumulative SES frameworks. We showed that social mobility was related with a functional lung state in adult life. To our knowledge, only two study has attempted to analyze SES trajectories during the life course in relation to lung function disparities. Menezes et al. have showed that SES at birth, in adolescence, and its trajectory between these two periods were inversely associated with lung function measurement by $FEV_1$and FVC in Brazilians[39]. Ramsey et al. have showed that combined adverse socioeconomic position in both adult life and childhood was additionally associated with a greater decline in lung function in men[34].

Within the cumulative SES score, our results showed that the accumulation of social disadvantage is linked to poorer lung function in women; however, we did not find this pattern in men. After adjustment for adult SES-related covariates, graded associations with pulmonary measurements were attenuated, which suggests that childhood conditions are less important in men and it supports the significance of contemporary SES-related factors.

The mechanism of the association between SES and lung function is not fully understood. The biological pathway by which a limited lung development in prenatal period is related to infant respiratory infection and higher sensitivity to impaired lung function in adult is relatively well established. Another explanation is the sociobiological pathway: SES in childhood is adversely associated with postnatal lung function, which causes worse lung development as well as impact on the immune system through increased exposure to risk factors like air pollution, passive smoking, or poor nutrition. Respiratory diseases in childhood may be an obstacle in gaining the right education, which leads to lower SES in adulthood.

Differences in the respiratory system functioning among adults with certain SES may be explained by differences in the occupation - especially the intensity and frequency of physically hazardous work that has a direct impact on the respiratory system[34]. In addition, low SES is

For peer review only - http://bmjopen.bmj.com/site/about/guidelines.xhtml

associated with more frequent exposure to risk factors, such as nutrition disorders, smoking, more frequent infections, and obstructive pulmonary disease[37,40-42].

All the above factors are simultaneously related with the SES. That is why the most possible explanation for the relation between SES and the deterioration of respiratory functions is multifactorial due to simultaneous influence of many factors, which enables to explain, partially at least, the described relations.

Our study introduced some new aspects to current knowledge. Firstly, this study is the first in CEE, which considers SES in the light of social and political transformations after the fall of the communist system. Secondly, we have defined model-based measures of childhood SES and adulthood SES that capture the multidimensionality of SES, which provide a detailed picture of the defined SES levels. This novel approach might facilitate the understanding of complexity of SES construct. For example, while about 60% of men from high SES childhood group had attained the university education, which would place them in higher SES group taking education as a single SES indicator, this percentage dropped to 47% for those belonging to high SES group assessed as composite measure. This marked the inconsistency in SES groups, which can be explained in two ways. First, levels of education do not go together with other dimensions of SES. Second, current economic situation (one of the elements of our SES composite measure) that provides resources to control one's circumstances overcomes the educational level among people who are nearly 60 years old and live in this part of Europe.

However, there are some limitations in the interpretation of the results. First, the cross-sectional study design does not allow address the causality of the observed relations. SES and, especially, its economical components are sensitive to changes over time. This may influence the health and then support health selection hypothesis, which in turn would strengthen the relation between SES and the studied health outcome. Secondly, information on childhood SES was based on a distant recall of various markers of social circumstances (up to 59 years in the oldest study participants). The retrospective nature of this of information increases the random error and it is subject to recall bias. The known effect of the overestimation of socioeconomic position in childhood, assessed on the of about father's/mother's education, might also bias the results[43].

For peer review only - http://bmjopen.bmj.com/site/about/guidelines.xhtml

Conclusion

Low SES over life course was associated with the lowest lung function. Downward social mobility was associated with poorer pulmonary function while upward mobility or life course moderate or high SES were associated with better pulmonary function.

For peer review only

For peer review only - http://bmjopen.bmj.com/site/about/guidelines.xhtml

Funding:

The author(s) has disclosed the receipt of the following financial support for the research, authorship, and/or publication of this article: this work was funded by the Welcome Trust (grant WT064947 and WT081081), the US National Institute of Aging (grant R01 AG23522), and the MacArthur Foundation.

Contribution:

Maciej Polak drafted the manuscript, designed the analysis and did data analysis.
Krystyna Szafraniec contributed to the data analysis and helped draft manuscript, critical revised the manuscript.
Magdalena Kozela was responsible for critical revision of the manuscript.
Renata Wolfhaut-Wolak was responsible for tables preparations and critical revision of the manuscript.
Martin Bobak contributed to the study design, critical revision of the manuscript.
Andrzej Pająk contributed to the study design, critical revision of the manuscript.
All authors approved the final version of the manuscript and this submission

Data sharing statement:

Data not in public domain.

Competing Interest Statement:

All authors report grants from The Welcome Trust, grants from The US National Institute of Aging, grants from The MacArthur Foundation during the conduct of the study. AP: personal fees from AMGEN and Sanofi, not related with the submitted work.

For peer review only - http://bmjopen.bmj.com/site/about/guidelines.xhtml

References

1 Kunst A. Socioeconomic inequalities in health in Central and Eastern Europe: synthesis of results of eight new studies. *Int J Public Health* 2009;54:197–200.

2 Mackenbach JP, Stirbu I, Roskam AJR, et al. Socioeconomic inequalities in health in 22 European countries. *New Engl J Med* 2008;358:2468–81.

3 Mackenbach JP, Huisman M, Andersen O, et al. Inequalities in lung cancer mortality by the educational level in 10 European populations. *Eur J Cancer* 2004;40:126–35.

4 Adler NE, Boyce T, Chesney MA, et al. Socioeconomic status and health: the challenge of the gradient. *Am. Psychol.* 1993;49:15–24.

5 Braveman PA, Cubbin C, Egerter S, et al. Socioeconomic disparities in health in the United States: What the patterns tell us. *Am J Public Health*, 2010;(Suppl 1),186–96

6 Stringhini S, Batty GD, Bovet , et al. Association of Lifecourse Socioeconomic Status with Chronic Inflammation and Type 2 Diabetes Risk: The Whitehall II Prospective Cohort Study. *PLoS Med* 2013

7 Galobardes B, Shaw M, Lawlor DA, et al. Indicators of socioeconomic position (part 1). Journal of Epidemiology & Community Health 2006;60:7-12.

8 Kuh D, Ben-Shlomo Y, Lynch J, Hallqvist J, Power C. Life course epidemiology. J Epidemiol Community Health. 2005;57:778–83

9 McEwen BS, Gianaros PJ. Central role of the brain in stress and adaptation: Links to socioeconomic status, health, and disease, *Ann N Y Acad Sci*. 2010;1186:190–222.

10 Richard PS, Huang M-H, Sidney S, et. al. Socioeconomic status and health: is parasympathetic nervous system activity an intervening mechanism? *Int J Epid* 2005;34:309–315

11 Fox AJ, Goldblatt PO, Jones DR. Social class mortality differentials: artefact, selection, or life circumstance. *J. Epidemiol. Commun. Health* 198; 39: 1–8.

12 Mulatu MS, Schooler C. Causal connections between socio-economic status and health: reciprocal effects and mediating mechanisms *J. Health Soc. Behav***.** 2002;43: 22-41

13 Galobardes B, Lynch J W, Smith G D. Childhood Socioeconomic Circumstances and Cause-specific Mortality in Adulthood: Systematic Review and Interpretation, *Epidemiologic Reviews*, Volume 26, Issue 1, 1 July 2004, Pages 7–21

14 Mayer KU. New directions in life course research. *Ann Rev Sociology 2009*;35:413–433.

15 Laurie M, Corna A. life course perspective on socioeconomic inequalities in health: A critical review of conceptual frameworks, *Adv Life Course Res***.** 2013,18:150–159

16 Cutler DM, Lleras-Muney A, Vogl T. Socioeconomic Status and Health: Dimensions and Mechanisms. Publication Date: Sep 2012.

17 Hallqvist J, Lynch J, Bartley M, Lang T, Blane D. Can we disentangle life course processes of accumulation, critical period and social mobility? An analysis of disadvantaged socio-economic positions and myocardial infarction in the Stockholm Heart Epidemiology Program. Soc Sci Med. 2004;58:1555–62

18 Marin TJ, Chen E, Miller GE. What Do Trajectories of Childhood Socioeconomic Status Tell Us About Markers of Cardiovascular Health in Adolescence? *Psychosom Med* 2008;70:152–159

For peer review only - http://bmjopen.bmj.com/site/about/guidelines.xhtml

19 Matthews KA, Kiefe CI, Lewis CE, et al. Socioeconomic Trajectories and Incident Hypertension in a Biracial Cohort of Young Adults. *Hypertension* 2002;39:772-776.

20 Kozyrskyj AL, Kendall GE, Jacoby P, Sly PD, et al. Association Between Socioeconomic Status and the Development of Asthma: Analyses of Income Trajectories. *Am J Public Health*. 2010;100:540–546.

21 Tabak C, Spijkerman AMW, Verschuren WMM, et al. Does educational level influence lung function decline (Doetinchem Cohort Study)? *Eur Respir J* 2009; 34:940–947

22 Schünemann HJ, Dorn J, Grant BJB, et al. Pulmonary Function Is a Long-term Predictor of Mortality in the General Population*: 29-Year Follow-up of the Buffalo Health Study *Chest* 2000;118(3):656-664.

23 Stavem K, Aaser E, Sandvik L, et al. Lung function, smoking and mortality in a 26-year follow-up of healthy middle-aged Males. *Eur Respir J* 2005; 25 618–625

24 Johannessen A, Eagan TML, Omenaas ER, et al. Socioeconomic risk factors for lung function decline in a general population A. *Eur Respir J* 2010;36:480–487

25 Peasey A, Bobak M, Kubinova R, et al. Determinants of cardiovascular disease and other non-communicable disease in Central and Eastern Europe: rationale and design of the HAPIEE study. BMC Public Health. 2006;6:255.

26 Miller MR, Hankinsosn J, Brusasco V, et al. Standardisation of spirometry. *Eur. Respir. J. 2005* 26;319-338.

27 Vandenheede H, Vikhireva O, Pikhart H, et al. Socioeconomic inequalities in all-cause mortality in the Czech Republic, Russia, Poland and Lithuania in the 2000s: findings from the HAPIEE Study. J Epidemiol Community Health 2014;68:297-303.

28 Ciu T, Fang D, Chen J, and Jerois , C. (2001). A Robust and Scalable Clustering Algorithm, for Mixed Type Attributes in Large Database Environment. *In: Proceedings of the seventh ACM SIGKDD international conference on knowledge discovery and data mining. San Francisco, CA: ACM 2001.*

29 Gray LA, Leyland AH, Benzeval M, et al. Explaining the social patterning of lung function in adulthood at different ages: the roles of childhood precursors, health behaviours and environmental factors. *J Epidemiol Community Health*. 2013;67(11):905–11.

30 McFadden E, Luben R, Wareham N, et al. How far can we explain the social class differential in respiratory function? A cross-sectional population study of 21,991 men and women from EPIC-Norfolk. *Eur J Epidemiol*. 2009;24(4):193–201

31 Merkus PJ, ten Have-Opbroek AA, Quanjer PH Human lung growth: areview. *Pediatr Pulmonol* 1996, 21(6):383-397.

32 Hegewald MJ, Crapo RO. Socioeconomic status and lung function. *Chest*. 2007;132(5):1608–14.

33 Gaffney AW, Hang J, Lee MS, et al. Socioeconomic status is associated with reduce lung function in China: an analysis from large cross-sectional study in Shanghai. *BMC Public Health* 2016;16:96

34 Ramsay SE, Whincup PH, Lennon LT, et al. Longitudinal associations of socioeconomic position in childhood and adulthood with decline in lung function over 20 years: results from a population-based cohort of British men. *Thorax*. 2011 Dec;66(12):1058-64.

35 Conry MC, Morgan K, Curry P, McGee H, Harrington J, Ward M, et al. The clustering of health behaviours in Ireland and their relationship with mental health, self-rated health and quality of life. BMC Public Health. 2011;11(1):692–701.

36 Clatworthy J, Hankins M, Buick D, et al. Cluster analysis in illness perception research: A Monte Carlo study to identify the most appropriate method. Psychology & Health 2007; 22:2,123-142,

For peer review only - http://bmjopen.bmj.com/site/about/guidelines.xhtml

37 Krzyzanowski M, Jedrychowski W. Occupational exposure and incidence of chronic respiratory symptoms among residents of Cracow followed for 13 years. Int Arch Occup Environ Health. 1990;62(4):311-7

38 Przewozniak K. , Sulkowska U, et al.. Tobacco smoking in countries of the European Union. Ann Agric Environ Med. 2012;19(2):181-92.

39 Menezes MB, Dumith SC, Perez-Padilla R, et al. Socioeconomic trajectory from birth to adolescence and lung function: prospective birth cohort study. *BMC Public Health*;2011,11:596

40 Shaheen SO, Jameson KA, Syddall HE, et al. The relationship of dietary patterns with adult lung function and COPD. *Eur Respir J*. 2010;36(2):277–84.

41Tabak C, Smit HA, Rasanen L, et al. Dietary factors and pulmonary function: a cross sectional study in middle aged men from three European countries. *Thorax*. 1999;54(11):1021–6.

42 Strachan DP. Ventilatory function, height, and mortality among lifelong non-smokers. *J Epidemiol Community Health* 1992; 46:66–70.

43 Batty GD, Lawlor DA, Macintyre S, et al. Accuracy of adults' recall of childhood social class: findings from the Aberdeen children of the 1950s study. *J Epidemiol Community Health* 2005;59:898-903.

For peer review only

STROBE Statement—checklist of items that should be included in reports of observational studies

	Item No	Recommendation
**Title and abstract**	1	Indicate the study's design with a commonly used term in the title or the abstract – page 1, lines: 2-3
		(*b*) Provide in the abstract an informative and balanced summary of what was done and what was found – page 2, lines :11-25
**Introduction**		
Background/rationale	2	Explain the scientific background and rationale for the investigation being reported – page 4, lines 2-8; page 5, lines: 12-18
Objectives	3	State specific objectives, including any prespecified hypotheses page 5, lines:12-18
**Methods**		
Study design	4	Present key elements of study design early in the paper – page 6, lines3-4
Setting	5	Describe the setting, locations, and relevant dates, including periods of recruitment, exposure, follow-up, and data collection – page 6, lines:5-9
Participants	6	(*a*) *Cohort study*—Give the eligibility criteria, and the sources and methods of selection of participants. Describe methods of follow-up<br>*Case-control study*—Give the eligibility criteria, and the sources and methods of case ascertainment and control selection. Give the rationale for the choice of cases and controls<br>*Cross-sectional study*—Give the eligibility criteria, and the sources and methods of selection of participants – page 6, lines: 5-8
		(*b*) *Cohort study*—For matched studies, give matching criteria and number of exposed and unexposed<br>*Case-control study*—For matched studies, give matching criteria and the number of controls per case
Variables	7	Clearly define all outcomes, exposures, predictors, potential confounders, and effect modifiers. Give diagnostic criteria, if applicable – page 6, lines:24-31, pages 7-8
Data sources/ measurement	8*	For each variable of interest, give sources of data and details of methods of assessment (measurement). Describe comparability of assessment methods if there is more than one group – pages 6-9
Bias	9	Describe any efforts to address potential sources of bias page – page 17, lines 26-28
Study size	10	Explain how the study size was arrived at – page 10, lines:6-9
Quantitative variables	11	Explain how quantitative variables were handled in the analyses. If applicable, describe which groupings were chosen and why – page 9, lines: 13-14
Statistical methods	12	(*a*) Describe all statistical methods, including those used to control for confounding– pages 9, lines: 13-31
		(*b*) Describe any methods used to examine subgroups and interactions – page 9, lines: 25-30
		(*c*) Explain how missing data were addressed– page 10, lines:6-7
		(*d*) *Cohort study*—If applicable, explain how loss to follow-up was addressed<br>*Case-control study*—If applicable, explain how matching of cases and controls was addressed<br>*Cross-sectional study*—If applicable, describe analytical methods taking account of sampling strategy
		(*e*) Describe any sensitivity analyses

For peer review only

**Results**		
Participants	13*	(a) Report numbers of individuals at each stage of study—eg numbers potentially eligible, examined for eligibility, confirmed eligible, included in the study, completing follow-up, and analysed – page 10, lines: 6-9
		(b) Give reasons for non-participation at each stage
		(c) Consider use of a flow diagram
Descriptive data	14*	(a) Give characteristics of study participants (eg demographic, clinical, social) and information on exposures and potential confounders – page 10, lines,; page 11, lines: 1-7
		(b) Indicate number of participants with missing data for each variable of interest – page 10, lines: 6-8
		(c) *Cohort study*—Summarise follow-up time (eg, average and total amount)
Outcome data	15*	*Cohort study*—Report numbers of outcome events or summary measures over time
		Case-control study—Report numbers in each exposure category, or summary measures of exposure
		Cross-sectional study—Report numbers of outcome events or summary measures – page 11, table
Main results	16	(*a*) Give unadjusted estimates and, if applicable, confounder-adjusted estimates and their precision (eg, 95% confidence interval). Make clear which confounders were adjusted for and why they were included – pages 12-13
		(*b*) Report category boundaries when continuous variables were categorized – not applicable
		(*c*) If relevant, consider translating estimates of relative risk into absolute risk for a meaningful time period
Other analyses	17	Report other analyses done—eg analyses of subgroups and interactions, and sensitivity analyses – page 10, lines 7-11
**Discussion**		
Key results	18	Summarise key results with reference to study objectives – page 15, lines 2-5
Limitations	19	Discuss limitations of the study, taking into account sources of potential bias or imprecision. Discuss both direction and magnitude of any potential bias – page 17, lines 20-28
Interpretation	20	Give a cautious overall interpretation of results considering objectives, limitations, multiplicity of analyses, results from similar studies, and other relevant evidence – page 14
Generalisability	21	Discuss the generalisability (external validity) of the study results– page 17, lines 7-19
**Other information**		
Funding	22	Give the source of funding and the role of the funders for the present study and, if applicable, for the original study on which the present article is based– page 19

*Give information separately for cases and controls in case-control studies and, if applicable, for exposed and unexposed groups in cohort and cross-sectional studies.

Note: An Explanation and Elaboration article discusses each checklist item and gives methodological background and published examples of transparent reporting. The STROBE checklist is best used in conjunction with this article (freely available on the Web sites of PLoS Medicine at http://www.plosmedicine.org/, Annals of Internal Medicine at http://www.annals.org/, and Epidemiology at http://www.epidem.com/). Information on the STROBE Initiative is available at www.strobe-statement.org.

For peer review only

BMJ Open

Socioeconomic status and pulmonary function, transition from childhood to adulthood: cross-sectional results from the Polish part of the HAPIEE study.

Journal:	*BMJ Open*
Manuscript ID	bmjopen-2018-022638.R2
Article Type:	Research
Date Submitted by the Author:	07-Oct-2018
Complete List of Authors:	Polak, Maciej; Jagiellonian University Medical College, Krakow, Poland, Chair of Epidemiology and Population Studies, Faculty of Health Sciences,<br>Szafraniec, Krystyna; Jagiellonian University Medical College, Krakow, Poland, Chair of Epidemiology and Population Studies, Faculty of Health Sciences<br>Kozela, Magdalena ; Jagiellonian University Medical College, Krakow, Poland, Chair of Epidemiology and Population Studies, Faculty of Health Sciences,<br>Wolfshaut-Wolak, Renata; Jagiellonian University Medical College, Krakow, Poland, Chair of Epidemiology and Population Studies, Faculty of Health Sciences<br>Bobak, Martin; University College London, Epidemiology and Public Health<br>Pająk, Andrzej; Jagiellonian University Medical College, Krakow, Poland, Chair of Epidemiology and Population Studies, Faculty of Health Sciences
**Primary Subject Heading**:	Epidemiology
Secondary Subject Heading:	Sociology
Keywords:	SES, Lung function, Social mobility

SCHOLARONE™
Manuscripts

Title:

Socioeconomic status and pulmonary function, transition from childhood to adulthood: cross-sectional results from the Polish part of the HAPIEE study

Maciej Polak, Krystyna Szafraniec, Magdalena Kozela, Renata Wolfshaut-Wolak, Martin Bobak, Andrzej Pająk

Affiliation: Chair of Epidemiology and Population Studies, Institute of Public Health, Jagiellonian University Medical College

Corresponding author:

Maciej Polak

Chair of Epidemiology and Population Studies
Faculty of Health Science
Jagiellonian University Medical College
31-531 Kraków, ul. Grzegórzecka 20
phone: +48 12 43 32 837
e-mail: maciej.1.polak@uj.edu.pl

For peer review only

Abstract

Objective

Previous studies have reported inverse associations between socioeconomic status (SES) and lung function but less is known about whether pulmonary function is affected by SES changes.

We aimed to describe the relationship of changes of SES between childhood and adulthood with pulmonary function.

Design

Cross-sectional study.

Participants

The study sample included 4104 men and women, aged 45–69 years, residents of Krakow, participating in the Polish part of the HAPIEE Project

Main outcome

Forced expiratory volume (FEV_1) and forced vital capacity (FVC) were assessed by the standardized spirometry procedure. Participants were classified into the 3 categories of SES (low, moderate, or high) based on information on parent's education, housing standard during childhood, own education, employment status, household amenities, and financial status.

Results

The adjusted difference in mean FVC between persons with low and high adulthood SES was 100 ml (p=0.005) in men and 100 ml (p<0.001) in women; the differences in mean FEV_1 were 103 ml (p<0.001) and 80 ml (p<0.001), respectively. Upward social mobility and moderate or high SES at both childhood and adulthood were related to significantly higher FEV_1 and FVC compared to low SES at both childhood and adulthood or downward social mobility.

Conclusions

Low SES over a life course was associated with the lowest lung function. Downward social mobility was associated with poorer pulmonary function, while upward mobility or life course and moderate or high SES were associated with better pulmonary function.

For peer review only - http://bmjopen.bmj.com/site/about/guidelines.xhtml

Strengths and limitations of this study.

Strengths

- This is a first study addressing the association between social mobility and pulmonary function in single urban population in Central Eastern Europe.
- The study sample was selected from the general population aged 45-69.
- Multidimensional nature of SES was explored.

Limitation

- The cross-sectional study design does not allow to address the causality of the observed relations.
- Distant recall of the markers of social circumstances could have contributed to overestimation of childhood SES.

For peer review only

For peer review only - http://bmjopen.bmj.com/site/about/guidelines.xhtml

Introduction

Socioeconomic status (SES) is one of the main predictors of health at each stage of the life cycle[1-3]. People in lower socioeconomic groups usually experience poorer health than people from higher socioeconomic groups, regardless of the health measures and SES indicators used[4-6]. SES is a complex, multidimensional social construct conceptualized to include income, education, and occupation (and other factors) which are often interrelated. However, it is likely that each SES measure reflects slightly different forces associated with health and disease on individual and societal levels[7].

Several social, psychological, and biological mechanisms have been proposed to explain the association of SES with health[8-10]. It has been suggested that SES cannot directly affect health as physiological variables do, but that it acts indirectly through behavioral factors or chronic stress that determine health[8]. In addition, people of higher socioeconomic status are also more likely to utilize knowledge, financial resources and beneficial social networks in order to evaluate risk factors and take up protective actions. On the other hand, there is also evidence for the reverse relationship between SES and health: e.g. the social drift hypothesis states that poor health leads to a downward shift in social class[11-12].

Studies that focus on determinants of adult disease frequently ignore the influence of factors from previous stages of life. Standard practice for measuring SES in most health studies is to include measures of current socioeconomic characteristics. Even if childhood socioeconomic position is measured, it is usually accounted for in multivariable models without taking into account the temporal relationship between variables[13]. The life course approach to how SES affects health has been increasingly applied to studying health[14-16]. The life course framework consists of several, partially interrelated models. The most frequently used concepts regarding the influence of life course SES on health are: 1/ the critical period model emphasizing the importance of the timing of SES exposure, 2/ the social mobility model considering the influence of changes in markers of SES over time followed by upward mobility or downward mobility, which in turn may impact health, 3/ the cumulative exposure model which hypothesizes that factors acting at different stages of life accumulate to influence disease risk in later life[9,17]. Thus, the life course approach allows to go beyond simple measures of childhood and adulthood SES to better capture important aspects of SES exposure for disease development. Whilst the life course approach has already been implemented to study the effects of SES on CVD[18], obesity[5], hypertension[19], and diabetes[6], it has been applied less frequently to respiratory diseases[20,21].

For peer review only - http://bmjopen.bmj.com/site/about/guidelines.xhtml

Lung function is a long-term predictor of morbidity and mortality from a range of diseases including non-respiratory conditions[22,23]. Studies have demonstrated the association between a decrease in lung function and not only increasing age, but also low SES[24]. However, little is known about how changes in SES patterns influence the respiratory health. None of the research from Central and Eastern Europe (CEE) has examined this association and hence this topic becomes challenging. This is especially because of the political transformation of 1989/90 occurring in this region, which was followed by rapid social and economic changes, resulting in the widening of income inequalities and changes in health behavior. Additionally, most middle-aged people's life crossed over that time period resulting in some phenomena that childhood SES was well established before and adulthood SES have been developing after the fall of socialism.

The aim of this study was to examine the socioeconomic gradient in pulmonary function in middle aged adults. We hypothesized that social mobility is positively related to pulmonary function measured by forced vital capacity (FVC) and one-second forced expiratory volume (FEV_1). The analysis was guided by three research questions, all aimed at characterizing the socioeconomic patterning of lung functions after adjustment for modifiable risk factors such as smoking and body mass index (BMI): (1) whether there is a gradual (dose-related) association between childhood and adulthood SES and pulmonary function measured by FVC and FEV_1; (2) whether there is a relationship between social mobility and pulmonary function; and (3) whether there is a relationship between cumulative SES and pulmonary function.

For peer review only - http://bmjopen.bmj.com/site/about/guidelines.xhtml

Methods

Study design and data collection

The analysis was performed using data from the Polish part of the HAPIEE (*Health, Alcohol and Psychosocial Factors In Eastern Europe*) study. The design of this study has been described in detail elsewhere[25]. In summary, the study included four urban cohorts selected from population registers in Novosibirsk (Russia), Krakow (Poland), six Czech towns and Kaunas (Lithuania); each consisted of a random sample of men and women aged 45–69 years at baseline in 2002-2005. All participants completed a comprehensive health questionnaire and underwent a brief medical examination including a pulmonary function test. In Poland, trained nurses interviewed the respondents at their homes. For this analysis information about age, current and past socioeconomic circumstances, health status (asthma or history of respiratory disease), and health behavior, i.e., smoking and physical activity was obtained using a standard questionnaire. Then, all participants were invited to a clinic for anthropometric and blood pressure measurements followed by blood collection for biochemical tests and for performance of spirometry.

Patient and Public Involvement statement

Participants were not involved in the design of this study.

Measures

Pulmonary function assessment

In our analysis, we used two parameters of pulmonary function: FVC, which is recognized as a predictor of vital status; and FEV_1, which is regarded as a good biological marker of risk of obstructive pulmonary disease. FVC and FEV_1 were measured by a trained technician in accordance with a standardized protocol[26] using a Micro-Medical Microplus spirometer. Participants were asked to make at least three maximal expiratory measures. If the quality of the spirometry was not satisfactory, the maneuver was repeated until the best quality was obtained. The highest value of FVC and the highest value of FEV_1 were chosen from those measurements for which the repeatability criteria were met. The quality of all tests was reviewed by a pulmonologist. Criteria for a satisfactory repeatability were based on the European Respiratory Society recommendations[26]. Exclusion criteria for the spirometry

For peer review only - http://bmjopen.bmj.com/site/about/guidelines.xhtml

included any chest surgery during previous three months, any physician-diagnosed serious cardiac condition (stroke, myocardial infarction), and recent respiratory infections.

Socioeconomic Status

Childhood SES was assessed using the level of education of both parents (incomplete primary or no formal education, primary, vocational, secondary, or university) and information about housing standard at the age of about 10 (cold tap water, hot tap water, radio, fridge, own kitchen, and own toilet), all summed up into an index measuring the amenities in childhood.

Adulthood SES was measured by five items: the study of the participant's educational attainment, professional activity, household amenities, and current financial situation. The participant's education was assessed by questioning his/her highest level of completed education (incomplete primary or no formal education, primary, vocational, secondary, or university). Current employment status encompassed the following categories: entrepreneur, employed and self-employed, unemployed including housewife, and retired people. An index of household amenities was constructed by summing up the number of valuable items that a participant had in his/her household (microwave, washing machine, video recorder, color television, dishwasher, freezer, camcorder, satellite TV, telephone and mobile phone). Current financial status was assessed by two questions: "How often does it happen not to have enough money for food which you and your family need?" and "Do you have any difficulties with paying bills?". The possible answers were the following: all the time, often, sometimes, rarely, or never. The same indicators were used in other reports from the HAPIEE study[27].

A two-step clustering algorithm was applied to classify participants into homogeneous groups in respect of childhood SES and adulthood SES. In our analysis we allowed the process to identify the dimensions of SES without constraint to any particular number of factors, in order to capture the natural complexity of SES. Three groups of childhood SES were generated, assessing reasonable evidence of the cluster structure (silhouette coefficient s(i)=0.51). Participants who had poor housing conditions (low value of childhood amenities index) and whose parents had no formal education or had at most completed primary school were classified by the algorithm as a low SES group. The middle SES group comprised subjects characterized by medium housing conditions in childhood and with secondary education have been completed by at least one of the participant's patents. A high childhood

For peer review only - http://bmjopen.bmj.com/site/about/guidelines.xhtml

amenities index value and parent with secondary or university education classified a participant into the high childhood SES group. Similarly, three groups of adult SES were identified (s(i)=0.55). Participants who had a university education, were employed, and had a high index of amenities were classified by the cluster algorithm as having high SES in adulthood. On the other hand, participants with low educational achievements, unemployed, being on a retirement pension, or reporting a lack of money for food or paying bills were classified into a low SES group. The middle SES group comprised participants mainly characterized having completed secondary education (however, 44% had a primary education), half-and-half working and retired persons having no problem with money for food and paying bills regularly (**Table 1**).

Table 1. Definition of the socioeconomic status

Socioeconomic status		Descriptive statistics	Cluster description
Childhood	Low	n=1632<br>Age (years): 58.9<br>Female (%):51	- Primary parent’s education<br>- Low value on amenities index in childhood
	Middle	n=1463<br>Age (years): 58.4<br>Female (%): 51	- Primary or secondary parent's education<br>- Low or middle value on amenities index in childhood
	High	n=881<br>Age (years): 56.0<br>Female (%): 50	- Secondary or university parent's education<br>- High value on amenities index in childhood
Adulthood	Low	n=1313<br>Age (years): 57.8<br>Female (%): 58	- Primary or secondary education<br>- Often or sometimes do not have enough money for food or have problems with paying bills<br>- Unemployed or pensioner
	Middle	n=1588<br>Age (years): 58.9<br>Female (%): 49	- Primary or secondary education<br>- Rarely or never do not have enough insufficient money for food or have problems with paying bills
	High	n=1101<br>Age (years): 57.1<br>Female (%): 44	- University education<br>- Employed or owner of company<br>- High value on amenities index

Cumulative SES score was calculated by summing the childhood and adulthood SES evaluations obtained by cluster analysis with code 0 for low SES, code 1 for medium SES and code 2 for high SES. The final cumulative SES score was further categorized as low (score=0–1), medium (score=2–3), and high (score=4).

For peer review only - http://bmjopen.bmj.com/site/about/guidelines.xhtml

Transition from childhood to adulthood (social mobility)

Having separate estimates for early childhood SES and adult SES allowed each participant to be allocated to one of the following four separate groups of SES changes: participants who were classified in the low subgroups in both time points were categorized as “always low”; those who were classified in the low SES in childhood, but who in adulthood were middle or high, were categorized as “upward mobility”. Similarly, those classified in middle or high SES in childhood and low in adulthood were categorized as “downward mobility”. The categories moderate and high SES were combined due to the similar differences in pulmonary function of participants who changed SES from low to moderate and from low to high SES. Finally, those classified in both life stages as having middle or high SES were categorized as “always moderate or high”.

Statistical analysis

Descriptive statistics stratified by sex are presented as means and standard deviations for pulmonary function parameters and other continuous measurements and as frequency (n, %) for categorical variables. Two-step cluster analysis was used to determine homogenous subgroups of childhood SES and adult SES. The procedure is particularly designed to handle large data set and allows consideration of both continuous and categorical variables. In the first step the algorithm uses a sequential clustering method to allocate the cases into many small sub-clusters. The second step takes sub-clusters resulting from the first step as input in order to group them into clusters. In the modeling process, the hierarchical clustering method and the log-likelihood distance measure between clusters were used. The validation of consistency within clusters of data was based on the silhouette coefficient s(i). Values above 0.5 are recognized as either reasonable or strong evidence of cluster structure (details are briefly described in[28]).

To compare FVC and FEV_1, and the distribution by SES in childhood, in adulthood, and in transition of SES between these two periods, a set of analyses of covariance were performed separately for men and women. Due to the ordinal character of the SES variables, p-values for linear trends in mean values of spirometry parameters (FEV_1 and FVC, separately) are presented. The final models were adjusted for age, height, BMI, smoking, and the prevalence

For peer review only - http://bmjopen.bmj.com/site/about/guidelines.xhtml

of respiratory diseases. Data was analyzed using IBM® SPSS® ver.22. All tests were two-tailed, and a p-value less than 0.05 was considered statistically significant.

For peer review only

For peer review only - http://bmjopen.bmj.com/site/about/guidelines.xhtml

Results

Table 2. Characteristics of the studied sample by sex

	Male n=2020		Female n=2084		p
	mean	SD	mean	SD	
Age	57.9	6.86	57.2	6.962	0.001[A]
BMI	28.10	4.130	28.47	5.040	0.016[A]
FEV_1 [L]	3.15	0.659	2.27	0.473	<0.001[A]
FVC [L]	3.81	0.717	2.73	5.280	<0.001[A]
	n	%	n	%	
Respiratory diseases (yes)	266	13.4	285	13.8	0.6[B]
Smoking status					
Current smoker	666	33.1	539	25.9	
Ex-smoker	755	37.5	419	20.2	<0.001[B]
Never smoker	592	29.4	1121	53.9	
Adulthood SES					
Low	548	27.1	765	36.7	
Middle	804	39.8	784	37.6	<0.001[B]
High	614	30.4	487	23.9	
Childhood SES					
Low	799	40.7	833	41.3	
Middle	723	36.9	740	36.7	0.9[B]
High	439	22.4	442	21.9	
Social mobility					
Always low	225	11.7	314	15.9	
Downward mobility	308	16.1	424	21.5	<0.001[B]
Upward mobility	557	29.1	503	25.5	
Always moderate or high	825	43.1	732	37.1	
Cumulative SES score					
Low	833	43.5	996	50.5	
Middle	729	43.3	769	39	<0.001[B]
High	253	13.2	208	10.5	

A–p value for t-test
B–p value for chi-square test

Out of the 4,840 participants for whom spirometry measurements were performed, 4,104 (84.8%) participants provided valid spirometry data and were included in this analysis. There were no differences in age, BMI, smoking, and SES level in childhood between the analyzed group and excluded participants. But, the latter group had a higher prevalence of respiratory diseases and a higher percentage (by 7%) of low SES category in adulthood. The characteristics of the study sample are presented in **Table 2**. The mean age was 55.6

For peer review only - http://bmjopen.bmj.com/site/about/guidelines.xhtml

(SD=6.92) years; men were slightly older than women (57.9 years vs. 57.2 years). Mean FEV_1 for the whole sample was 2.70 L (95% CI 2.68 to 2.73), men had a 0.88 L higher mean FEV_1 than women. Mean FVC was 3.25 L (95% CI 3.22 to 3.28), the difference between men and women in mean FVC was statistically significant and was equal to 1.08 L. The distribution of SES indicators (adulthood, cumulative, and transition) differed between sexes. Low SES in adulthood was observed in 27% of men and 37% of women ($p<0.001$). No differences in the distribution of childhood SES emerged from sex groups.

Tables 3 and **4** show means of FEV_1 and FVC, related to SES indices (childhood SES, adulthood SES, cumulative SES and social mobility), in the model adjusted for age and the model adjusted for all other covariates. Age adjusted means of FEV_1 and FVC were associated with childhood (in women), adulthood, and cumulative SES score with a significant gradient in men and women. Compared to high SES, men in the low or middle SES had significantly worse lung function (differences ranging from 0.20 L to 0.27 L in FEV_1 and 0.10 L to 0.26 L in FVC). Results were similar but less pronounced in women (differences from 0.07 L to 0.16 L in case of FEV_1 and 0.18 L to 0.25 L in FVC). Controlling for age, height, BMI, smoking, and the prevalence of respiratory diseases resulted in some attenuation of the estimates, but the pattern and significance of the association remained, except for the relationship between childhood SES and FVC, as well as cumulative SES and FEV_1 and FVC in men.

For peer review only - http://bmjopen.bmj.com/site/about/guidelines.xhtml

Table 3. **Means of FEV_1 [L] by category of childhood, adulthood SES, transition SES, and cumulative assessment of SES.**

	Male				Female			
	model A		model B		model A		model B	
Adulthood SES	mean	95% CI	mean	95% CI	men	95% CI	mean	95% CI
Low	3.01	(2.96-3.06)	2.9	(2.85-2.94)	2.22	(2.19-2.25)	2.12	(2.09-2.15)
Middle	3.16	(3.12-3.20)	2.99	(2.95-3.03)	2.27	(2.24-2.30)	2.15	(2.12-2.18)
High	3.28	(3.24-3.32)	3.03	(2.98-3.07)	2.36	(2.33-2.40)	2.2	(2.16-2.24)
p for trend	<0.001		<0.001		<0.001		0.02	
Childhood SES	mean	95% CI	mean	95% CI	mean	95% CI	mean	95% CI
Low	3.15	(3.11-3.19)	3.00	(2.95-3.04)	2.27	(2.24-2.29)	2.16	(2.13-2.19)
Middle	3.18	(3.08-3.27)	2.95	(2.90-2.99)	2.25	(2.22-2.28)	2.12	(2.09-2.16)
High	3.19	(3.14-3.25)	2.94	(2.89-3.00)	2.34	(2.30-2.37)	2.19	(2.15-2.23)
p for trend	0.1		0.1		0.001		0.011	
Cumulative SES score	mean	95% CI	mean	95% CI	mean	95% CI	mean	95% CI
Low	3.09	(3.05-3.13)	2.95	(2.91-3.00)	2.24	(2.18-2.24)	2.14	(2.11-2.17)
Middle	3.18	(3.14-3.22)	2.97	(2.93-3.01)	2.29	(2.26-2.32)	2.15	(2.12-2.18)
High	3.29	(3.22-3.36)	2.99	(2.92-3.06)	2.4	(2.35-2.46)	2.23	(2.17-2.28)
p for trend	<0.001		0.6		<0.001		0.009	
Social mobility		95% CI	mean	95% CI	mean	95% CI	mean	95% CI
Always low	2.98	(2.90-3.05)	2.91	(2.84-2.98)	2.21	(2.17-2.26)	2.14	(2.10-2.19)
Downward mobility	3.03	(2.97-3.10)	2.88	(2.82-2.94)	2.23	(2.19-2.27)	2.13	(2.09-2.17)
Upward mobility	3.21	(3.16-3.26)	3.03	(2.98-3.08)	2.3	(2.27-2.34)	2.19	(2.15-2.23)
Always moderate or high	3.21	(3.17-3.24)	3.04	(2.98-3.07)	2.31	(2.28-2.34)	2.21	(2.18-2.24)
p for trend	<0.001		<0.001		<0.001		0.048	

model A—adjusted for age

model B—adjusted for age, height, BMI, smoking status and respiratory disease.

For peer review only

For peer review only - http://bmjopen.bmj.com/site/about/guidelines.xhtml

Table 4. **Means of FVC [L] by category of childhood, adulthood, and transition SES and cumulative assessment of SES.**

	Male				Female			
	model A		model B			model A	model B	
Adulthood SES	mean	95% CI	mean	95% CI	mean	95% CI	mean	95% CI
Low	3.66	(3.61-3.72)	3.61	(3.55-3.67)	2.67	(2.64-2.71)	2.63	(2.59-3.66)
Middle	3.83	(3.78-3.88)	3.73	(3.68-3.78)	2.71	(2.67-2.74)	2.65	(2.61-2.69)
High	3.92	(3.86-3.97)	3.71	(3.65-3.76)	2.85	(2.81-2.89)	2.73	(2.68-2.77)
p for trend	<0.001		0.005		<0.001			<0.001
Childhood SES	mean	95% CI	mean	95% CI	mean	95% CI	mean	95% CI
Low	3.79	(3.74-3.83)	3.71	(3.66-3.76)	2,69	(2.66-2.72)	2.642	(2.61-2.68)
Middle	3.77	(3.72-3.82)	3.66	(3.60-3.71)	2.71	(2.68-2.75)	2.635	(2.60-2.67)
High	3.89	(3.83-3.96)	3.69	(3.62-3.75)	2.84	(2.79-2.88)	2.725	(2.68-2.77)
p for trend	0.001		0.1		<0.001		0.001	
Cumulative SES score	mean	95% CI	mean	95% CI	mean	95% CI	mean	95% CI
Low	3.73	(3.67-3.78)	3.67	(3.62-3.72)	2.67	(2.64-2.70)	2.63	(2.60-2.67)
Middle	3.84	(3.80-3.89)	3.69	(3.64-3.74)	2.76	(2.72-2.79)	2.66	(2.62-2.70)
High	3.93	(3.84-4.02)	3.67	(3.58-3.75)	2.92	(2.85-2.98)	2.78	(2.71-2.84)
p for trend	<0.001		0.7		<0.001		<0.01	
Social mobility		95% CI	mean	95% CI	mean	95% CI	mean	95% CI
Always low	3.59	(3.50-3.68)	3.6	(3.52-3.67)	2.64	(2.58-2.69)	2.62	(2.57-2.67)
Downward mobility	3.72	(3.64-3.80)	3.63	(3.56-3.70)	2.71	(2.66-2.75)	2.64	(2.59-2.68)
Upward mobility	3.87	(3.81-3.92)	3.77	(3.71-3.83)	2.72	(2.68-2.76)	2.67	(2.63-2.72)
Always moderate or high	3.86	(3.81-3.91)	3.79	(3.74-3.8 5)	2.8	(2.76-2.83)	2.69	(2.66-2.73)
p for trend	<0.001		<0.001		<0.001			0.02

model A—adjusted for age

model B—adjusted for age, height, BMI, smoking status, and respiratory disease.

For peer review only - http://bmjopen.bmj.com/site/about/guidelines.xhtml

Social mobility was related to both FEV_1 and FVC. In men and women, participants whose SES decreased to low had pulmonary function similar to participants who always had low SES. Compared to persons with life course low SES or SES decreased to low, persons with life course moderate or high SES or increased SES, had higher FVC and FEV_1. The differences between participants who always had low SES compared to those with moderate or high SES were 190 ml for FVC and 130 ml for FEV_1, in men. In women the differences were smaller and equal to 70 ml for both FVC and FEV_1 (**Tables 3–4**).

For peer review only

For peer review only - http://bmjopen.bmj.com/site/about/guidelines.xhtml

Discussion

The study showed that moderate or high childhood and adulthood SES, as well as upward social mobility were associated with better pulmonary function i.e. higher means of FEV_1 and FVC. The relationship was attenuated slightly, particularly in men after adjustment for age, height, smoking, obesity, and history of respiratory diseases but remained statistically significant. The attenuation was mostly explained by height, which itself serves as a marker of early-life disadvantage and directly affects the lung function[29-31].

Results from our study, with reference to life stages of childhood or adulthood SES, are consistent with previous studies[21,30-34], which have shown that pulmonary function is gradually worse in the lower SES groups, although the magnitude of the effect varies across studies.

In this study, we applied a novel approach to determine childhood SES and adulthood SES from the range of socioeconomic indicators. Almost all previous studies used either indicator of SES (education, income, or occupation) or attempted to explain the independent effect of one indicator, by adjusting for other indices in regression analyses. However, this approach is questioned as it does not take into account the joint effect of these factors and their interrelationship[16]. In our study, we used a model-based framework (cluster analysis) to identify SES groups that captured a detailed heterogeneity in education, previous and current economic situation, and current occupational status. Cluster analysis is not a traditional approach to social stratification, although it was applied in some studies[35-36]. This method seems to be particularly suitable for CEE society in which social classes are less clearly separated from each other. As a result, we observed that for the adult SES, low and middle SES did not differ in terms of education and home amenities index. They differed significantly in their current occupational status, as the low SES group was dominated by pensioners and unemployed people, which further, partially, explained the differences in current material resources for everyday living. The associations between the complexity of SES and pulmonary functions were in line with other studies[30-34].

Considering childhood SES, parent's educational achievements overlap slightly between low-middle as well as middle-high SES groups reflecting the fact that a single indicator of education does not sufficiently capture the complex pattern of SES characteristics. However, the standard of housing (the second component of SES) separated the low and high groups unequivocally. In contrast to other studies, we did not find significant low–high SES differences

For peer review only - http://bmjopen.bmj.com/site/about/guidelines.xhtml

in pulmonary measurements in men, after adjustments for adult risk factors. This suggests that the SES effect in childhood might be masked by SES in adulthood. Although parent's education significantly determines children's educational opportunity, during the socialist period the common national policy was to equalize the chances for education by favoring to children from lower socioeconomic groups. This policy reduced the inequality in education; however, because of rapid industrialization of the country, education was strongly vocationally oriented. Hazardous work and the prevalence of more frequent smoking in men may partially explain the lack of this significance[37-38].

The present study supported two life course hypotheses originating from the social mobility and cumulative SES frameworks. We showed that social mobility was related with a functional lung state in adult life. To our knowledge, only two studies have attempted to analyze SES trajectories during life course in relation to lung function disparities. Menezes et al. showed that SES at birth, in adolescence, and its trajectory between these two periods was inversely associated with lung function measurements by $FEV_1$and FVC in Brazilians[39]. Ramsey et al. showed that combination of adverse socioeconomic position in both adult life and childhood was additionally associated with a greater decline in lung function in men[34].

Within the cumulative SES score, our results showed that the accumulation of social disadvantage is linked to poorer lung function in women; however, we did not find this pattern in men. After adjustment for adult SES-related covariates, graded associations with pulmonary measurements were attenuated, which suggests that childhood conditions are less important in men and this supports the significance of contemporary SES-related factors.

The mechanism of the association between SES and lung function is not fully understood. The biological pathway by which limited lung development in the prenatal period is related to infant respiratory infection and higher sensitivity to impaired lung function in adulthood is relatively well established. Another explanation is the sociobiological pathway: SES in childhood is adversely associated with postnatal lung function, which leads to worse lung development as well as an impact on the immune system through increased exposure to risk factors such as air pollution, passive smoking, or poor nutrition. Respiratory diseases in childhood may be an obstacle in gaining the right education, which leads to lower SES in adulthood.

Differences in respiratory system functioning among adults with certain SES may be explained by differences in occupation especially the intensity and frequency of physically

For peer review only - http://bmjopen.bmj.com/site/about/guidelines.xhtml

hazardous work that has a direct impact on the respiratory system[34]. In addition, low SES is associated with more frequent exposure to risk factors, such as nutrition disorders, smoking, more frequent infections, and obstructive pulmonary disease[37,40-42].

All the above factors are simultaneously related with the SES. That is why the most possible explanation for the relationship between SES and the deterioration of respiratory functions is multifactorial due to the simultaneous influence of many factors. This would enable an explanation, a partial one at least, of the described relationships.

Our study has introduced some new aspects to current knowledge. Firstly, this study is the first in CEE which considers SES in the light of social and political transformations after the fall of the communist system. Secondly, we have defined model-based measures of childhood SES and adulthood SES that capture the multidimensionality of SES, which provide a detailed picture of the defined SES levels. This novel approach might facilitate the understanding of the complexity of the SES construct. For example, while about 60% of men from the high SES childhood group had a university education, which would place them in higher the SES group when taking education as a single SES indicator, this percentage dropped to 47% for those belonging to the high SES group, when assessed as a composite measure. This highlighted the inconsistency in SES groups, which can be explained in two ways. First, levels of education do not fit together with other dimensions of SES. Second, the current economic situation (one of the elements of our SES composite measure) that provides resources to control one's circumstances overcomes the educational level among people who are nearly 60 years old and live in this part of Europe.

However, there are some limitations to the interpretation of the results. First, the cross-sectional study design does not allow address the causality of the observed relations. SES, and especially its economical components are sensitive to changes over time. This may influence the health and then support health selection hypothesis, which in turn would strengthen the relationship between SES and the studied health outcome. Secondly, information on childhood SES was based on the distant recall of various markers of social circumstances (up to 59 years in the oldest study participants). The retrospective nature of this of information increases the random error and it is subject to recall bias. The known effect of the overestimation of socioeconomic position in childhood, assessed on the basis of parental education might also bias the results[43]. Finally, broader generalization of the results is limited as the cluster analysis which was used to assess the SES categories is sensitive to the nature of the data, types of relationships between variables and the methods of grouping cases into distinct clusters.

For peer review only - http://bmjopen.bmj.com/site/about/guidelines.xhtml
For peer review only

Conclusion

Low SES over a life course was associated with the lowest lung function. Downward social mobility was associated with poorer pulmonary function while upward mobility or life course moderate or high SES were associated with better pulmonary function.

For peer review only

For peer review only - http://bmjopen.bmj.com/site/about/guidelines.xhtml

Funding:

The author(s) has disclosed the receipt of the following financial support for the research, authorship, and/or publication of this article: this work was funded by the Wellcome Trust (grant WT064947 and WT081081), the US National Institute of Aging (grant R01 AG23522), and the MacArthur Foundation.

Contribution:

Maciej Polak drafted the manuscript, designed the analysis and performed the data analysis.
Krystyna Szafraniec contributed to the data analysis and helped draft the manuscript, critical revised the manuscript.
Magdalena Kozela was responsible for critical revision of the manuscript.
Renata Wolfhaut-Wolak was responsible for tables preparations and critical revision of the manuscript.
Martin Bobak contributed to the study design, and critical revision of the manuscript.
Andrzej Pająk contributed to the study design, and critical revision of the manuscript.
All authors approved the final version of the manuscript and this submission

Data sharing statement:

Data not in public domain.

Competing Interest Statement:

All the authors report grants from The Wellcome Trust, grants from the US National Institute of Aging, grants from The MacArthur Foundation during the conduct of the study. AP: personal fees from AMGEN and Sanofi, not related with the submitted work.

For peer review only - http://bmjopen.bmj.com/site/about/guidelines.xhtml

References

1 Kunst A. Socioeconomic inequalities in health in Central and Eastern Europe: synthesis of results of eight new studies. *Int J Public Health* 2009;54:197–200.

2 Mackenbach JP, Stirbu I, Roskam AJR, et al. Socioeconomic inequalities in health in 22 European countries. *New Engl J Med* 2008;358:2468–81.

3 Mackenbach JP, Huisman M, Andersen O, et al. Inequalities in lung cancer mortality by the educational level in 10 European populations. *Eur J Cancer* 2004;40:126–35.

4 Adler NE, Boyce T, Chesney MA, et al. Socioeconomic status and health: the challenge of the gradient. *Am. Psychol.* 1993;49:15–24.

5 Braveman PA, Cubbin C, Egerter S, et al. Socioeconomic disparities in health in the United States: What the patterns tell us. *Am J Public Health*, 2010;(Suppl 1),186–96

6 Stringhini S, Batty GD, Bovet , et al. Association of Lifecourse Socioeconomic Status with Chronic Inflammation and Type 2 Diabetes Risk: The Whitehall II Prospective Cohort Study. *PLoS Med* 2013

7 Galobardes B, Shaw M, Lawlor DA, et al. Indicators of socioeconomic position (part 1). Journal of Epidemiology & Community Health 2006;60:7-12.

8 Kuh D, Ben-Shlomo Y, Lynch J, Hallqvist J, Power C. Life course epidemiology. J Epidemiol Community Health. 2005;57:778–83

9 McEwen BS, Gianaros PJ. Central role of the brain in stress and adaptation: Links to socioeconomic status, health, and disease, *Ann N Y Acad Sci*. 2010;1186:190–222.

10 Richard PS, Huang M-H, Sidney S, et. al. Socioeconomic status and health: is parasympathetic nervous system activity an intervening mechanism? *Int J Epid* 2005;34:309–315

11 Fox AJ, Goldblatt PO, Jones DR. Social class mortality differentials: artefact, selection, or life circumstance. *J. Epidemiol. Commun. Health* 198; 39: 1–8.

12 Mulatu MS, Schooler C. Causal connections between socio-economic status and health: reciprocal effects and mediating mechanisms *J. Health Soc. Behav.* 2002;43: 22-41

13 Galobardes B, Lynch J W, Smith G D. Childhood Socioeconomic Circumstances and Cause-specific Mortality in Adulthood: Systematic Review and Interpretation, *Epidemiologic Reviews*, Volume 26, Issue 1, 1 July 2004, Pages 7–21

14 Mayer KU. New directions in life course research. *Ann Rev Sociology 2009*;35:413–433.

15 Laurie M, Corna A. life course perspective on socioeconomic inequalities in health: A critical review of conceptual frameworks, *Adv Life Course Res*. 2013,18:150–159

16 Cutler DM, Lleras-Muney A, Vogl T. Socioeconomic Status and Health: Dimensions and Mechanisms. Publication Date: Sep 2012.

17 Hallqvist J, Lynch J, Bartley M, Lang T, Blane D. Can we disentangle life course processes of accumulation, critical period and social mobility? An analysis of disadvantaged socio-economic positions and myocardial infarction in the Stockholm Heart Epidemiology Program. Soc Sci Med. 2004;58:1555–62

18 Marin TJ, Chen E, Miller GE. What Do Trajectories of Childhood Socioeconomic Status Tell Us About Markers of Cardiovascular Health in Adolescence? *Psychosom Med* 2008;70:152–159

For peer review only - http://bmjopen.bmj.com/site/about/guidelines.xhtml

19 Matthews KA, Kiefe CI, Lewis CE, et al. Socioeconomic Trajectories and Incident Hypertension in a Biracial Cohort of Young Adults. *Hypertension* 2002;39:772-776.

20 Kozyrskyj AL, Kendall GE, Jacoby P, Sly PD, et al. Association Between Socioeconomic Status and the Development of Asthma: Analyses of Income Trajectories. *Am J Public Health*. 2010;100:540–546.

21 Tabak C, Spijkerman AMW, Verschuren WMM, et al. Does educational level influence lung function decline (Doetinchem Cohort Study)? *Eur Respir J* 2009; 34:940–947

22 Schünemann HJ, Dorn J, Grant BJB, et al. Pulmonary Function Is a Long-term Predictor of Mortality in the General Population*: 29-Year Follow-up of the Buffalo Health Study *Chest* 2000;118(3):656-664.

23 Stavem K, Aaser E, Sandvik L, et al. Lung function, smoking and mortality in a 26-year follow-up of healthy middle-aged Males. *Eur Respir J* 2005; 25 618–625

24 Johannessen A, Eagan TML, Omenaas ER, et al. Socioeconomic risk factors for lung function decline in a general population A. *Eur Respir J* 2010;36:480–487

25 Peasey A, Bobak M, Kubinova R, et al. Determinants of cardiovascular disease and other non-communicable disease in Central and Eastern Europe: rationale and design of the HAPIEE study. BMC Public Health. 2006;6:255.

26 Miller MR, Hankinsosn J, Brusasco V, et al. Standardisation of spirometry. *Eur. Respir. J. 2005* 26;319-338.

27 Vandenheede H, Vikhireva O, Pikhart H, et al. Socioeconomic inequalities in all-cause mortality in the Czech Republic, Russia, Poland and Lithuania in the 2000s: findings from the HAPIEE Study. J Epidemiol Community Health 2014;68:297-303.

28 Ciu T, Fang D, Chen J, and Jerois , C. (2001). A Robust and Scalable Clustering Algorithm, for Mixed Type Attributes in Large Database Environment. *In: Proceedings of the seventh ACM SIGKDD international conference on knowledge discovery and data mining. San Francisco, CA: ACM 2001.*

29 Gray LA, Leyland AH, Benzeval M, et al. Explaining the social patterning of lung function in adulthood at different ages: the roles of childhood precursors, health behaviours and environmental factors. *J Epidemiol Community Health*. 2013;67(11):905–11.

30 McFadden E, Luben R, Wareham N, et al. How far can we explain the social class differential in respiratory function? A cross-sectional population study of 21,991 men and women from EPIC-Norfolk. *Eur J Epidemiol*. 2009;24(4):193–201

31 Merkus PJ, ten Have-Opbroek AA, Quanjer PH Human lung growth: areview. *Pediatr Pulmonol* 1996, 21(6):383-397.

32 Hegewald MJ, Crapo RO. Socioeconomic status and lung function. *Chest*. 2007;132(5):1608–14.

33 Gaffney AW, Hang J, Lee MS, et al. Socioeconomic status is associated with reduce lung function in China: an analysis from large cross-sectional study in Shanghai. *BMC Public Health* 2016;16:96

34 Ramsay SE, Whincup PH, Lennon LT, et al. Longitudinal associations of socioeconomic position in childhood and adulthood with decline in lung function over 20 years: results from a population-based cohort of British men. *Thorax*. 2011 Dec;66(12):1058-64.

35 Conry MC, Morgan K, Curry P, McGee H, Harrington J, Ward M, et al. The clustering of health behaviours in Ireland and their relationship with mental health, self-rated health and quality of life. BMC Public Health. 2011;11(1):692–701.

36 Clatworthy J, Hankins M, Buick D, et al. Cluster analysis in illness perception research: A Monte Carlo study to identify the most appropriate method. Psychology & Health 2007; 22:2,123-142,

For peer review only - http://bmjopen.bmj.com/site/about/guidelines.xhtml

37 Krzyzanowski M, Jedrychowski W. Occupational exposure and incidence of chronic respiratory symptoms among residents of Cracow followed for 13 years. Int Arch Occup Environ Health. 1990;62(4):311-7

38 Przewozniak K. , Sulkowska U, et al.. Tobacco smoking in countries of the European Union. Ann Agric Environ Med. 2012;19(2):181-92.

39 Menezes MB, Dumith SC, Perez-Padilla R, et al. Socioeconomic trajectory from birth to adolescence and lung function: prospective birth cohort study. *BMC Public Health*;2011,11:596

40 Shaheen SO, Jameson KA, Syddall HE, et al. The relationship of dietary patterns with adult lung function and COPD. *Eur Respir J*. 2010;36(2):277–84.

41Tabak C, Smit HA, Rasanen L, et al. Dietary factors and pulmonary function: a cross sectional study in middle aged men from three European countries. *Thorax*. 1999;54(11):1021–6.

42 Strachan DP. Ventilatory function, height, and mortality among lifelong non-smokers. *J Epidemiol Community Health* 1992; 46:66–70.

43 Batty GD, Lawlor DA, Macintyre S, et al. Accuracy of adults' recall of childhood social class: findings from the Aberdeen children of the 1950s study. *J Epidemiol Community Health* 2005;59:898-903.

For peer review only

STROBE Statement—checklist of items that should be included in reports of observational studies

	Item No	Recommendation
**Title and abstract**	1	Indicate the study's design with a commonly used term in the title or the abstract – page 1, lines: 2-3
		(*b*) Provide in the abstract an informative and balanced summary of what was done and what was found – page 2, lines :11-25
**Introduction**		
Background/rationale	2	Explain the scientific background and rationale for the investigation being reported – page 4, lines 2-8; page 5, lines: 12-18
Objectives	3	State specific objectives, including any prespecified hypotheses page 5, lines:12-18
**Methods**		
Study design	4	Present key elements of study design early in the paper – page 6, lines3-4
Setting	5	Describe the setting, locations, and relevant dates, including periods of recruitment, exposure, follow-up, and data collection – page 6, lines:5-9
Participants	6	(*a*) *Cohort study*—Give the eligibility criteria, and the sources and methods of selection of participants. Describe methods of follow-up<br>*Case-control study*—Give the eligibility criteria, and the sources and methods of case ascertainment and control selection. Give the rationale for the choice of cases and controls<br>*Cross-sectional study*—Give the eligibility criteria, and the sources and methods of selection of participants – page 6, lines: 5-8
		(*b*) *Cohort study*—For matched studies, give matching criteria and number of exposed and unexposed<br>*Case-control study*—For matched studies, give matching criteria and the number of controls per case
Variables	7	Clearly define all outcomes, exposures, predictors, potential confounders, and effect modifiers. Give diagnostic criteria, if applicable – page 6, lines:24-31, pages 7-8
Data sources/ measurement	8*	For each variable of interest, give sources of data and details of methods of assessment (measurement). Describe comparability of assessment methods if there is more than one group – pages 6-9
Bias	9	Describe any efforts to address potential sources of bias page – page 17, lines 26-28
Study size	10	Explain how the study size was arrived at – page 10, lines:6-9
Quantitative variables	11	Explain how quantitative variables were handled in the analyses. If applicable, describe which groupings were chosen and why – page 9, lines: 13-14
Statistical methods	12	(*a*) Describe all statistical methods, including those used to control for confounding– pages 9, lines: 13-31
		(*b*) Describe any methods used to examine subgroups and interactions – page 9, lines: 25-30
		(*c*) Explain how missing data were addressed– page 10, lines:6-7
		(*d*) *Cohort study*—If applicable, explain how loss to follow-up was addressed<br>*Case-control study*—If applicable, explain how matching of cases and controls was addressed<br>*Cross-sectional study*—If applicable, describe analytical methods taking account of sampling strategy
		(*e*) Describe any sensitivity analyses

For peer review only

**Results**		
Participants	13*	(a) Report numbers of individuals at each stage of study—eg numbers potentially eligible, examined for eligibility, confirmed eligible, included in the study, completing follow-up, and analysed – page 10, lines: 6-9
		(b) Give reasons for non-participation at each stage
		(c) Consider use of a flow diagram
Descriptive data	14*	(a) Give characteristics of study participants (eg demographic, clinical, social) and information on exposures and potential confounders – page 10, lines,; page 11, lines: 1-7
		(b) Indicate number of participants with missing data for each variable of interest – page 10, lines: 6-8
		(c) *Cohort study*—Summarise follow-up time (eg, average and total amount)
Outcome data	15*	*Cohort study*—Report numbers of outcome events or summary measures over time
		Case-control study—Report numbers in each exposure category, or summary measures of exposure
		Cross-sectional study—Report numbers of outcome events or summary measures – page 11, table
Main results	16	(*a*) Give unadjusted estimates and, if applicable, confounder-adjusted estimates and their precision (eg, 95% confidence interval). Make clear which confounders were adjusted for and why they were included – pages 12-13
		(*b*) Report category boundaries when continuous variables were categorized – not applicable
		(*c*) If relevant, consider translating estimates of relative risk into absolute risk for a meaningful time period
Other analyses	17	Report other analyses done—eg analyses of subgroups and interactions, and sensitivity analyses – page 10, lines 7-11
**Discussion**		
Key results	18	Summarise key results with reference to study objectives – page 15, lines 2-5
Limitations	19	Discuss limitations of the study, taking into account sources of potential bias or imprecision. Discuss both direction and magnitude of any potential bias – page 17, lines 20-28
Interpretation	20	Give a cautious overall interpretation of results considering objectives, limitations, multiplicity of analyses, results from similar studies, and other relevant evidence – page 14
Generalisability	21	Discuss the generalisability (external validity) of the study results– page 17, lines 7-19
**Other information**		
Funding	22	Give the source of funding and the role of the funders for the present study and, if applicable, for the original study on which the present article is based– page 19

*Give information separately for cases and controls in case-control studies and, if applicable, for exposed and unexposed groups in cohort and cross-sectional studies.

Note: An Explanation and Elaboration article discusses each checklist item and gives methodological background and published examples of transparent reporting. The STROBE checklist is best used in conjunction with this article (freely available on the Web sites of PLoS Medicine at http://www.plosmedicine.org/, Annals of Internal Medicine at http://www.annals.org/, and Epidemiology at http://www.epidem.com/). Information on the STROBE Initiative is available at www.strobe-statement.org.

For peer review only - http://bmjopen.bmj.com/site/about/guidelines.xhtml

For peer review only

BMJ Open

Socioeconomic status and pulmonary function, transition from childhood to adulthood: cross-sectional results from the Polish part of the HAPIEE study.

Journal:	*BMJ Open*
Manuscript ID	bmjopen-2018-022638.R3
Article Type:	Research
Date Submitted by the Author:	29-Oct-2018
Complete List of Authors:	Polak, Maciej; Jagiellonian University Medical College, Krakow, Poland, Chair of Epidemiology and Population Studies, Faculty of Health Sciences,<br>Szafraniec, Krystyna; Jagiellonian University Medical College, Krakow, Poland, Chair of Epidemiology and Population Studies, Faculty of Health Sciences<br>Kozela, Magdalena ; Jagiellonian University Medical College, Krakow, Poland, Chair of Epidemiology and Population Studies, Faculty of Health Sciences,<br>Wolfshaut-Wolak, Renata; Jagiellonian University Medical College, Krakow, Poland, Chair of Epidemiology and Population Studies, Faculty of Health Sciences<br>Bobak, Martin; University College London, Epidemiology and Public Health<br>Pająk, Andrzej; Jagiellonian University Medical College, Krakow, Poland, Chair of Epidemiology and Population Studies, Faculty of Health Sciences
**Primary Subject Heading**:	Epidemiology
Secondary Subject Heading:	Sociology
Keywords:	SES, Lung function, Social mobility

SCHOLARONE™
Manuscripts

Title:
Socioeconomic status and pulmonary function, transition from childhood to adulthood: cross-sectional results from the Polish part of the HAPIEE study

Maciej Polak, Krystyna Szafraniec, Magdalena Kozela, Renata Wolfshaut-Wolak, Martin Bobak, Andrzej Pająk

Affiliation: Chair of Epidemiology and Population Studies, Institute of Public Health, Jagiellonian University Medical College

Corresponding author:

Maciej Polak

Chair of Epidemiology and Population Studies
Faculty of Health Science
Jagiellonian University Medical College
31-531 Kraków, ul. Grzegórzecka 20
phone: +48 12 43 32 837
e-mail: maciej.1.polak@uj.edu.pl

For peer review only

Abstract

Objective

Previous studies have reported inverse associations between socioeconomic status (SES) and lung function but less is known about whether pulmonary function is affected by SES changes.

We aimed to describe the relationship of changes of SES between childhood and adulthood with pulmonary function.

Design

Cross-sectional study.

Participants

The study sample included 4104 men and women, aged 45–69 years, residents of Krakow, participating in the Polish part of the HAPIEE Project

Main outcome

Forced expiratory volume (FEV_1) and forced vital capacity (FVC) were assessed by the standardized spirometry procedure. Participants were classified into the 3 categories of SES (low, moderate, or high) based on information on parent's education, housing standard during childhood, own education, employment status, household amenities, and financial status.

Results

The adjusted difference in mean FVC between persons with low and high adulthood SES was 100 ml ($p=0.005$) in men and 100 ml ($p<0.001$) in women; the differences in mean FEV_1 were 103 ml ($p<0.001$) and 80 ml ($p<0.001$), respectively. Upward social mobility and moderate or high SES at both childhood and adulthood were related to significantly higher FEV_1 and FVC compared to low SES at both childhood and adulthood or downward social mobility.

Conclusions

Low SES over a life course was associated with the lowest lung function. Downward social mobility was associated with poorer pulmonary function, while upward mobility or life course and moderate or high SES were associated with better pulmonary function.

For peer review only - http://bmjopen.bmj.com/site/about/guidelines.xhtml

Strengths and limitations of this study.

Strengths

- This is a first study addressing the association between social mobility and pulmonary function in single urban population in Central Eastern Europe.
- The study sample was selected from the general population aged 45-69.
- Multidimensional nature of SES was explored.

Limitation

- The cross-sectional study design does not allow to address the causality of the observed relations.
- Distant recall of the markers of social circumstances could have contributed to overestimation of childhood SES.

For peer review only

For peer review only - http://bmjopen.bmj.com/site/about/guidelines.xhtml

Introduction

Socioeconomic status (SES) is one of the main predictors of health at each stage of the life cycle[1-3]. People in lower socioeconomic groups usually experience poorer health than people from higher socioeconomic groups, regardless of the health measures and SES indicators used[4-6]. SES is a complex, multidimensional social construct conceptualized to include income, education, and occupation (and other factors) which are often interrelated. However, it is likely that each SES measure reflects slightly different forces associated with health and disease on individual and societal levels[7].

Several social, psychological, and biological mechanisms have been proposed to explain the association of SES with health[8-10]. It has been suggested that SES cannot directly affect health as physiological variables do, but that it acts indirectly through behavioral factors or chronic stress that determine health[8]. In addition, people of higher socioeconomic status are also more likely to utilize knowledge, financial resources and beneficial social networks in order to evaluate risk factors and take up protective actions. On the other hand, there is also evidence for the reverse relationship between SES and health: e.g. the social drift hypothesis states that poor health leads to a downward shift in social class[11-12].

Studies that focus on determinants of adult disease frequently ignore the influence of factors from previous stages of life. Standard practice for measuring SES in most health studies is to include measures of current socioeconomic characteristics. Even if childhood socioeconomic position is measured, it is usually accounted for in multivariable models without taking into account the temporal relationship between variables[13]. The life course approach to how SES affects health has been increasingly applied to studying health[14-16]. The life course framework consists of several, partially interrelated models. The most frequently used concepts regarding the influence of life course SES on health are: 1/ the critical period model emphasizing the importance of the timing of SES exposure, 2/ the social mobility model considering the influence of changes in markers of SES over time followed by upward mobility or downward mobility, which in turn may

For peer review only - http://bmjopen.bmj.com/site/about/guidelines.xhtml

For peer review only

impact health, 3/ the cumulative exposure model which hypothesizes that factors acting at different stages of life accumulate to influence disease risk in later life[9,17]. Thus, the life course approach allows to go beyond simple measures of childhood and adulthood SES to better capture important aspects of SES exposure for disease development. Whilst the life course approach has already been implemented to study the effects of SES on CVD[18], obesity[5], hypertension[19], and diabetes[6], it has been applied less frequently to respiratory diseases[20,21].

Lung function is a long-term predictor of morbidity and mortality from a range of diseases including non-respiratory conditions[22,23]. Studies have demonstrated the association between a decrease in lung function and not only increasing age, but also low SES[24]. However, little is known about how changes in SES patterns influence the respiratory health. None of the research from Central and Eastern Europe (CEE) has examined this association and hence this topic becomes challenging. This is especially because of the political transformation of 1989/90 occurring in this region, which was followed by rapid social and economic changes, resulting in the widening of income inequalities and changes in health behavior. Additionally, most middle-aged people's life crossed over that time period resulting in some phenomena that childhood SES was well established before and adulthood SES have been developing after the fall of socialism.

The aim of this study was to examine the socioeconomic gradient in pulmonary function in middle aged adults. We hypothesized that social mobility is positively related to pulmonary function measured by forced vital capacity (FVC) and one-second forced expiratory volume (FEV_1). The analysis was guided by three research questions, all aimed at characterizing the socioeconomic patterning of lung functions after adjustment for modifiable risk factors such as smoking and body mass index (BMI): (1) whether there is a gradual (dose-related) association between childhood and adulthood SES and pulmonary function measured by FVC and FEV_1; (2) whether there is a relationship between social mobility and pulmonary function; and (3) whether there is a relationship between cumulative SES and pulmonary function.

For peer review only - http://bmjopen.bmj.com/site/about/guidelines.xhtml

Methods

Study design and data collection

The analysis was performed using data from the Polish part of the HAPIEE (*Health, Alcohol and Psychosocial Factors In Eastern Europe*) study. The design of this study has been described in detail elsewhere[25]. In summary, the study included four urban cohorts selected from population registers in Novosibirsk (Russia), Krakow (Poland), six Czech towns and Kaunas (Lithuania); each consisted of a random sample of men and women aged 45–69 years at baseline in 2002-2005. All participants completed a comprehensive health questionnaire and underwent a brief medical examination including a pulmonary function test. In Poland, trained nurses interviewed the respondents at their homes. For this analysis information about age, current and past socioeconomic circumstances, health status (asthma or history of respiratory disease), and health behavior, i.e., smoking and physical activity was obtained using a standard questionnaire. Then, all participants were invited to a clinic for anthropometric and blood pressure measurements followed by blood collection for biochemical tests and for performance of spirometry. The study was approved by the Bioethics Committee of the Jagiellonian University and by the University College London/University College London Hospital ethics committee. All participants gave informed consent for attendance in the study.

Patient and Public Involvement statement

Participants were not involved in the design of this study.

Measures

Pulmonary function assessment

In our analysis, we used two parameters of pulmonary function: FVC, which is recognized as a predictor of vital status; and FEV_1, which is regarded as a good biological marker of risk of obstructive pulmonary disease. FVC and FEV_1 were measured by a trained technician in accordance with a standardized protocol[26] using a Micro-Medical Microplus spirometer. Participants were asked to make at least three maximal expiratory measures. If the quality of the spirometry was not satisfactory, the maneuver was repeated until the best quality was obtained. The highest value of FVC and the highest value of FEV_1 were chosen from those measurements for which the repeatability criteria were met. The quality of all tests was reviewed by a pulmonologist. Criteria for a satisfactory repeatability were based on the European Respiratory

For peer review only - http://bmjopen.bmj.com/site/about/guidelines.xhtml

Society recommendations[26]. Exclusion criteria for the spirometry included any chest surgery during previous three months, any physician-diagnosed serious cardiac condition (stroke, myocardial infarction), and recent respiratory infections.

Socioeconomic Status

Childhood SES was assessed using the level of education of both parents (incomplete primary or no formal education, primary, vocational, secondary, or university) and information about housing standard at the age of about 10 (cold tap water, hot tap water, radio, fridge, own kitchen, and own toilet), all summed up into an index measuring the amenities in childhood.

Adulthood SES was measured by five items: the study of the participant's educational attainment, professional activity, household amenities, and current financial situation. The participant's education was assessed by questioning his/her highest level of completed education (incomplete primary or no formal education, primary, vocational, secondary, or university). Current employment status encompassed the following categories: entrepreneur, employed and self-employed, unemployed including housewife, and retired people. An index of household amenities was constructed by summing up the number of valuable items that a participant had in his/her household (microwave, washing machine, video recorder, color television, dishwasher, freezer, camcorder, satellite TV, telephone and mobile phone). Current financial status was assessed by two questions: "How often does it happen not to have enough money for food which you and your family need?" and "Do you have any difficulties with paying bills?". The possible answers were the following: all the time, often, sometimes, rarely, or never. The same indicators were used in other reports from the HAPIEE study[27].

A two-step clustering algorithm was applied to classify participants into homogeneous groups in respect of childhood SES and adulthood SES. In our analysis we allowed the process to identify the dimensions of SES without constraint to any particular number of factors, in order to capture the natural complexity of SES. Three groups of childhood SES were generated, assessing reasonable evidence of the cluster structure (silhouette coefficient s(i)=0.51). Participants who had poor housing conditions (low value of childhood amenities index) and whose parents had no formal education or had at most completed primary school were classified by the algorithm as a low SES group. The middle SES group comprised subjects characterized by medium housing conditions in

For peer review only - http://bmjopen.bmj.com/site/about/guidelines.xhtml

childhood and with secondary education have been completed by at least one of the participant's patents. A high childhood amenities index value and parent with secondary or university education classified a participant into the high childhood SES group. Similarly, three groups of adult SES were identified (s(i)=0.55). Participants who had a university education, were employed, and had a high index of amenities were classified by the cluster algorithm as having high SES in adulthood. On the other hand, participants with low educational achievements, unemployed, being on a retirement pension, or reporting a lack of money for food or paying bills were classified into a low SES group. The middle SES group comprised participants mainly characterized having completed secondary education (however, 44% had a primary education), half-and-half working and retired persons having no problem with money for food and paying bills regularly (**Table 1**).

Table 1. Definition of the socioeconomic status

Socioeconomic status		Descriptive statistics	Cluster description
Childhood	Low	n=1632<br>Age (years): 58.9<br>Female (%):51	- Primary parent's education<br>- Low value on amenities index in childhood
	Middle	n=1463<br>Age (years): 58.4<br>Female (%): 51	- Primary or secondary parent's education<br>- Low or middle value on amenities index in childhood
	High	n=881<br>Age (years): 56.0<br>Female (%): 50	- Secondary or university parent's education<br>- High value on amenities index in childhood
Adulthood	Low	n=1313<br>Age (years): 57.8<br>Female (%): 58	- Primary or secondary education<br>- Often or sometimes do not have enough money for food or have problems with paying bills<br>- Unemployed or pensioner
	Middle	n=1588<br>Age (years): 58.9<br>Female (%): 49	- Primary or secondary education<br>- Rarely or never do not have enough insufficient money for food or have problems with paying bills
	High	n=1101<br>Age (years): 57.1<br>Female (%): 44	- University education<br>- Employed or owner of company<br>- High value on amenities index

For peer review only - http://bmjopen.bmj.com/site/about/guidelines.xhtml

Cumulative SES score was calculated by summing the childhood and adulthood SES evaluations obtained by cluster analysis with code 0 for low SES, code 1 for medium SES and code 2 for high SES. The final cumulative SES score was further categorized as low (score=0–1), medium (score=2–3), and high (score=4).

Transition from childhood to adulthood (social mobility)

Having separate estimates for early childhood SES and adult SES allowed each participant to be allocated to one of the following four separate groups of SES changes: participants who were classified in the low subgroups in both time points were categorized as “always low”; those who were classified in the low SES in childhood, but who in adulthood were middle or high, were categorized as “upward mobility”. Similarly, those classified in middle or high SES in childhood and low in adulthood were categorized as “downward mobility”. The categories moderate and high SES were combined due to the similar differences in pulmonary function of participants who changed SES from low to moderate and from low to high SES. Finally, those classified in both life stages as having middle or high SES were categorized as “always moderate or high”.

Statistical analysis

Descriptive statistics stratified by sex are presented as means and standard deviations for pulmonary function parameters and other continuous measurements and as frequency (n, %) for categorical variables. Two-step cluster analysis was used to determine homogenous subgroups of childhood SES and adult SES. The procedure is particularly designed to handle large data set and allows consideration of both continuous and categorical variables. In the first step the algorithm uses a sequential clustering method to allocate the cases into many small sub-clusters. The second step takes sub-clusters resulting from the first step as input in order to group them into clusters. In the modeling process, the hierarchical clustering method and the log-likelihood distance measure between clusters were used. The validation of consistency within clusters of data was based on the silhouette coefficient s(i). Values above 0.5 are recognized as either reasonable or strong evidence of cluster structure (details are briefly described in[28]).

To compare FVC and FEV_1, and the distribution by SES in childhood, in adulthood, and in transition of SES between these two periods, a set of analyses of covariance were performed

For peer review only - http://bmjopen.bmj.com/site/about/guidelines.xhtml

separately for men and women. Due to the ordinal character of the SES variables, p-values for linear trends in mean values of spirometry parameters (FEV_1 and FVC, separately) are presented. The final models were adjusted for age, height, BMI, smoking, and the prevalence of respiratory diseases. Data was analyzed using IBM® SPSS® ver.22. All tests were two-tailed, and a p-value less than 0.05 was considered statistically significant.

For peer review only

For peer review only - http://bmjopen.bmj.com/site/about/guidelines.xhtml

Results

Table 2. Characteristics of the studied sample by sex

	Male n=2020		Female n=2084		p
	mean	SD	mean	SD	
Age	57.9	6.86	57.2	6.962	0.001[A]
BMI	28.10	4.130	28.47	5.040	0.016[A]
FEV_1 [L]	3.15	0.659	2.27	0.473	<0.001[A]
FVC [L]	3.81	0.717	2.73	5.280	<0.001[A]
	n	%	n	%	
Respiratory diseases (yes)	266	13.4	285	13.8	0.6[B]
Smoking status					
Current smoker	666	33.1	539	25.9	
Ex-smoker	755	37.5	419	20.2	<0.001[B]
Never smoker	592	29.4	1121	53.9	
Adulthood SES					
Low	548	27.1	765	36.7	
Middle	804	39.8	784	37.6	<0.001[B]
High	614	30.4	487	23.9	
Childhood SES					
Low	799	40.7	833	41.3	
Middle	723	36.9	740	36.7	0.9[B]
High	439	22.4	442	21.9	
Social mobility					
Always low	225	11.7	314	15.9	
Downward mobility	308	16.1	424	21.5	<0.001[B]
Upward mobility	557	29.1	503	25.5	
Always moderate or high	825	43.1	732	37.1	
Cumulative SES score					
Low	833	43.5	996	50.5	
Middle	729	43.3	769	39	<0.001[B]
High	253	13.2	208	10.5	

A–p value for t-test
B–p value for chi-square test

Out of the 4,840 participants for whom spirometry measurements were performed, 4,104 (84.8%) participants provided valid spirometry data and were included in this analysis. There were no differences in age, BMI, smoking, and SES level in childhood between the analyzed group and

For peer review only - http://bmjopen.bmj.com/site/about/guidelines.xhtml

excluded participants. But, the latter group had a higher prevalence of respiratory diseases and a higher percentage (by 7%) of low SES category in adulthood. The characteristics of the study sample are presented in **Table 2**. The mean age was 55.6 (SD=6.92) years; men were slightly older than women (57.9 years vs. 57.2 years). Mean FEV_1 for the whole sample was 2.70 L (95% CI 2.68 to 2.73), men had a 0.88 L higher mean FEV_1 than women. Mean FVC was 3.25 L (95% CI 3.22 to 3.28), the difference between men and women in mean FVC was statistically significant and was equal to 1.08 L. The distribution of SES indicators (adulthood, cumulative, and transition) differed between sexes. Low SES in adulthood was observed in 27% of men and 37% of women ($p<0.001$). No differences in the distribution of childhood SES emerged from sex groups.

Tables 3 and **4** show means of FEV_1 and FVC, related to SES indices (childhood SES, adulthood SES, cumulative SES and social mobility), in the model adjusted for age and the model adjusted for all other covariates. Age adjusted means of FEV_1 and FVC were associated with childhood (in women), adulthood, and cumulative SES score with a significant gradient in men and women. Compared to high SES, men in the low or middle SES had significantly worse lung function (differences ranging from 0.20 L to 0.27 L in FEV_1 and 0.10 L to 0.26 L in FVC). Results were similar but less pronounced in women (differences from 0.07 L to 0.16 L in case of FEV_1 and 0.18 L to 0.25 L in FVC). Controlling for age, height, BMI, smoking, and the prevalence of respiratory diseases resulted in some attenuation of the estimates, but the pattern and significance of the association remained, except for the relationship between childhood SES and FVC, as well as cumulative SES and FEV_1 and FVC in men.

For peer review only - http://bmjopen.bmj.com/site/about/guidelines.xhtml

For peer review only

Table 3. **Means of FEV_1 [L] by category of childhood, adulthood SES, transition SES, and cumulative assessment of SES.**

	Male				Female			
	model A		model B		model A		model B	
Adulthood SES	mean	95% CI	mean	95% CI	men	95% CI	mean	95% CI
Low	3.01	(2.96-3.06)	2.9	(2.85-2.94)	2.22	(2.19-2.25)	2.12	(2.09-2.15)
Middle	3.16	(3.12-3.20)	2.99	(2.95-3.03)	2.27	(2.24-2.30)	2.15	(2.12-2.18)
High	3.28	(3.24-3.32)	3.03	(2.98-3.07)	2.36	(2.33-2.40)	2.2	(2.16-2.24)
p for trend	<0.001		<0.001		<0.001		0.02	
Childhood SES	mean	95% CI	mean	95% CI	mean	95% CI	mean	95% CI
Low	3.15	(3.11-3.19)	3.00	(2.95-3.04)	2.27	(2.24-2.29)	2.16	(2.13-2.19)
Middle	3.18	(3.08-3.27)	2.95	(2.90-2.99)	2.25	(2.22-2.28)	2.12	(2.09-2.16)
High	3.19	(3.14-3.25)	2.94	(2.89-3.00)	2.34	(2.30-2.37)	2.19	(2.15-2.23)
p for trend	0.1		0.1		0.001		0.011	
Cumulative SES score	mean	95% CI	mean	95% CI	mean	95% CI	mean	95% CI
Low	3.09	(3.05-3.13)	2.95	(2.91-3.00)	2.24	(2.18-2.24)	2.14	(2.11-2.17)
Middle	3.18	(3.14-3.22)	2.97	(2.93-3.01)	2.29	(2.26-2.32)	2.15	(2.12-2.18)
High	3.29	(3.22-3.36)	2.99	(2.92-3.06)	2.4	(2.35-2.46)	2.23	(2.17-2.28)
p for trend	<0.001		0.6		<0.001		0.009	
Social mobility		95% CI	mean	95% CI	mean	95% CI	mean	95% CI
Always low	2.98	(2.90-3.05)	2.91	(2.84-2.98)	2.21	(2.17-2.26)	2.14	(2.10-2.19)
Downward mobility	3.03	(2.97-3.10)	2.88	(2.82-2.94)	2.23	(2.19-2.27)	2.13	(2.09-2.17)
Upward mobility	3.21	(3.16-3.26)	3.03	(2.98-3.08)	2.3	(2.27-2.34)	2.19	(2.15-2.23)
Always moderate or high	3.21	(3.17-3.24)	3.04	(2.98-3.07)	2.31	(2.28-2.34)	2.21	(2.18-2.24)
p for trend	<0.001		<0.001		<0.001		0.048	

model A—adjusted for age

model B—adjusted for age, height, BMI, smoking status and respiratory disease.

For peer review only

Table 4. **Means of FVC [L] by category of childhood, adulthood, and transition SES and cumulative assessment of SES.**

	Male				Female			
	model A		model B		model A		model B	
Adulthood SES	mean	95% CI	mean	95% CI	mean	95% CI	mean	95% CI
Low	3.66	(3.61-3.72)	3.61	(3.55-3.67)	2.67	(2.64-2.71)	2.63	(2.59-3.66)
Middle	3.83	(3.78-3.88)	3.73	(3.68-3.78)	2.71	(2.67-2.74)	2.65	(2.61-2.69)
High	3.92	(3.86-3.97)	3.71	(3.65-3.76)	2.85	(2.81-2.89)	2.73	(2.68-2.77)
p for trend	<0.001		0.005			<0.001		<0.001
Childhood SES	mean	95% CI	mean	95% CI	mean	95% CI	mean	95% CI
Low	3.79	(3.74-3.83)	3.71	(3.66-3.76)	2,69	(2.66-2.72)	2.642	(2.61-2.68)
Middle	3.77	(3.72-3.82)	3.66	(3.60-3.71)	2.71	(2.68-2.75)	2.635	(2.60-2.67)
High	3.89	(3.83-3.96)	3.69	(3.62-3.75)	2.84	(2.79-2.88)	2.725	(2.68-2.77)
p for trend		0.001		0.1		<0.001		0.001
Cumulative SES score	mean	95% CI	mean	95% CI	mean	95% CI	mean	95% CI
Low	3.73	(3.67-3.78)	3.67	(3.62-3.72)	2.67	(2.64-2.70)	2.63	(2.60-2.67)
Middle	3.84	(3.80-3.89)	3.69	(3.64-3.74)	2.76	(2.72-2.79)	2.66	(2.62-2.70)
High	3.93	(3.84-4.02)	3.67	(3.58-3.75)	2.92	(2.85-2.98)	2.78	(2.71-2.84)
p for trend		<0.001		0.7		<0.001		<0.01
Social mobility		95% CI	mean	95% CI	mean	95% CI	mean	95% CI
Always low	3.59	(3.50-3.68)	3.6	(3.52-3.67)	2.64	(2.58-2.69)	2.62	(2.57-2.67)
Downward mobility	3.72	(3.64-3.80)	3.63	(3.56-3.70)	2.71	(2.66-2.75)	2.64	(2.59-2.68)
Upward mobility	3.87	(3.81-3.92)	3.77	(3.71-3.83)	2.72	(2.68-2.76)	2.67	(2.63-2.72)
Always moderate or high	3.86	(3.81-3.91)	3.79	(3.74-3.8 5)	2.8	(2.76-2.83)	2.69	(2.66-2.73)
p for trend		<0.001		<0.001		<0.001		0.02

model A—adjusted for age

model B—adjusted for age, height, BMI, smoking status, and respiratory disease.

For peer review only

Social mobility was related to both FEV_1 and FVC. In men and women, participants whose SES decreased to low had pulmonary function similar to participants who always had low SES. Compared to persons with life course low SES or SES decreased to low, persons with life course moderate or high SES or increased SES, had higher FVC and FEV_1. The differences between participants who always had low SES compared to those with moderate or high SES were 190 ml for FVC and 130 ml for FEV_1, in men. In women the differences were smaller and equal to 70 ml for both FVC and FEV_1 (**Tables 3–4**).

For peer review only

For peer review only - http://bmjopen.bmj.com/site/about/guidelines.xhtml

Discussion

The study showed that moderate or high childhood and adulthood SES, as well as upward social mobility were associated with better pulmonary function i.e. higher means of FEV_1 and FVC. The relationship was attenuated slightly, particularly in men after adjustment for age, height, smoking, obesity, and history of respiratory diseases but remained statistically significant. The attenuation was mostly explained by height, which itself serves as a marker of early-life disadvantage and directly affects the lung function[29-31].

Results from our study, with reference to life stages of childhood or adulthood SES, are consistent with previous studies[21,30-34], which have shown that pulmonary function is gradually worse in the lower SES groups, although the magnitude of the effect varies across studies.

In this study, we applied a novel approach to determine childhood SES and adulthood SES from the range of socioeconomic indicators. Almost all previous studies used either indicator of SES (education, income, or occupation) or attempted to explain the independent effect of one indicator, by adjusting for other indices in regression analyses. However, this approach is questioned as it does not take into account the joint effect of these factors and their interrelationship[16]. In our study, we used a model-based framework (cluster analysis) to identify SES groups that captured a detailed heterogeneity in education, previous and current economic situation, and current occupational status. Cluster analysis is not a traditional approach to social stratification, although it was applied in some studies[35-36]. This method seems to be particularly suitable for CEE society in which social classes are less clearly separated from each other. As a result, we observed that for the adult SES, low and middle SES did not differ in terms of education and home amenities index. They differed significantly in their current occupational status, as the low SES group was dominated by pensioners and unemployed people, which further, partially, explained the differences in current material resources for everyday living. The associations between the complexity of SES and pulmonary functions were in line with other studies[30-34].

Considering childhood SES, parent's educational achievements overlap slightly between low-middle as well as middle-high SES groups reflecting the fact that a single indicator of education does not sufficiently capture the complex pattern of SES characteristics. However, the standard of housing (the second component of SES) separated the low and high groups unequivocally. In contrast to other studies, we did not find significant low–high SES differences in pulmonary measurements in men,

For peer review only - http://bmjopen.bmj.com/site/about/guidelines.xhtml

after adjustments for adult risk factors. This suggests that the SES effect in childhood might be masked by SES in adulthood. Although parent's education significantly determines children's educational opportunity, during the socialist period the common national policy was to equalize the chances for education by favoring to children from lower socioeconomic groups. This policy reduced the inequality in education; however, because of rapid industrialization of the country, education was strongly vocationally oriented. Hazardous work and the prevalence of more frequent smoking in men may partially explain the lack of this significance[37-38].

The present study supported two life course hypotheses originating from the social mobility and cumulative SES frameworks. We showed that social mobility was related with a functional lung state in adult life. To our knowledge, only two studies have attempted to analyze SES trajectories during life course in relation to lung function disparities. Menezes et al. showed that SES at birth, in adolescence, and its trajectory between these two periods was inversely associated with lung function measurements by FEV_1 and FVC in Brazilians[39]. Ramsey et al. showed that combination of adverse socioeconomic position in both adult life and childhood was additionally associated with a greater decline in lung function in men[34].

Within the cumulative SES score, our results showed that the accumulation of social disadvantage is linked to poorer lung function in women; however, we did not find this pattern in men. After adjustment for adult SES-related covariates, graded associations with pulmonary measurements were attenuated, which suggests that childhood conditions are less important in men and this supports the significance of contemporary SES-related factors.

The mechanism of the association between SES and lung function is not fully understood. The biological pathway by which limited lung development in the prenatal period is related to infant respiratory infection and higher sensitivity to impaired lung function in adulthood is relatively well established. Another explanation is the sociobiological pathway: SES in childhood is adversely associated with postnatal lung function, which leads to worse lung development as well as an impact on the immune system through increased exposure to risk factors such as air pollution, passive smoking, or poor nutrition. Respiratory diseases in childhood may be an obstacle in gaining the right education, which leads to lower SES in adulthood.

Differences in respiratory system functioning among adults with certain SES may be explained by differences in occupation especially the intensity and frequency of physically hazardous work that has

For peer review only - http://bmjopen.bmj.com/site/about/guidelines.xhtml

a direct impact on the respiratory system[34]. In addition, low SES is associated with more frequent exposure to risk factors, such as nutrition disorders, smoking, more frequent infections, and obstructive pulmonary disease[37,40-42].

All the above factors are simultaneously related with the SES. That is why the most possible explanation for the relationship between SES and the deterioration of respiratory functions is multifactorial due to the simultaneous influence of many factors. This would enable an explanation, a partial one at least, of the described relationships.

Our study has introduced some new aspects to current knowledge. Firstly, this study is the first in CEE which considers SES in the light of social and political transformations after the fall of the communist system. Secondly, we have defined model-based measures of childhood SES and adulthood SES that capture the multidimensionality of SES, which provide a detailed picture of the defined SES levels. This novel approach might facilitate the understanding of the complexity of the SES construct. For example, while about 60% of men from the high SES childhood group had a university education, which would place them in higher the SES group when taking education as a single SES indicator, this percentage dropped to 47% for those belonging to the high SES group, when assessed as a composite measure. This highlighted the inconsistency in SES groups, which can be explained in two ways. First, levels of education do not fit together with other dimensions of SES. Second, the current economic situation (one of the elements of our SES composite measure) that provides resources to control one's circumstances overcomes the educational level among people who are nearly 60 years old and live in this part of Europe.

However, there are some limitations to the interpretation of the results. First, the cross-sectional study design does not allow address the causality of the observed relations. SES, and especially its economical components are sensitive to changes over time. This may influence the health and then support health selection hypothesis, which in turn would strengthen the relationship between SES and the studied health outcome. Secondly, information on childhood SES was based on the distant recall of various markers of social circumstances (up to 59 years in the oldest study participants). The retrospective nature of this of information increases the random error and it is subject to recall bias. The known effect of the overestimation of socioeconomic position in childhood, assessed on the basis of parental education might also bias the results[43]. Finally, broader generalization of the results is limited as the cluster analysis which was used to assess the SES categories is sensitive to the nature

For peer review only

For peer review only - http://bmjopen.bmj.com/site/about/guidelines.xhtml

of the data, types of relationships between variables and the methods of grouping cases into distinct clusters.

Conclusion

Low SES over a life course was associated with the lowest lung function. Downward social mobility was associated with poorer pulmonary function while upward mobility or life course moderate or high SES were associated with better pulmonary function.

For peer review only

For peer review only - http://bmjopen.bmj.com/site/about/guidelines.xhtml

Funding:

The author(s) has disclosed the receipt of the following financial support for the research, authorship, and/or publication of this article: this work was funded by the Wellcome Trust (grant WT064947 and WT081081), the US National Institute of Aging (grant R01 AG23522), and the MacArthur Foundation.

Contribution:

Maciej Polak drafted the manuscript, designed the analysis and performed the data analysis.
Krystyna Szafraniec contributed to the data analysis and helped draft the manuscript, critical revised the manuscript.
Magdalena Kozela was responsible for critical revision of the manuscript.
Renata Wolfhaut-Wolak was responsible for tables preparations and critical revision of the manuscript.
Martin Bobak contributed to the study design, and critical revision of the manuscript.
Andrzej Pająk contributed to the study design, and critical revision of the manuscript.
All authors approved the final version of the manuscript and this submission.

Data sharing statement:

Data not in public domain.

For peer review only - http://bmjopen.bmj.com/site/about/guidelines.xhtml

Competing Interest Statement:

All the authors report grants from The Wellcome Trust, grants from the US National Institute of Aging, grants from The MacArthur Foundation during the conduct of the study. AP: personal fees from AMGEN and Sanofi, not related with the submitted work.

For peer review only

For peer review only - http://bmjopen.bmj.com/site/about/guidelines.xhtml

References

1 Kunst A. Socioeconomic inequalities in health in Central and Eastern Europe: synthesis of results of eight new studies. *Int J Public Health* 2009;54:197–200.

2 Mackenbach JP, Stirbu I, Roskam AJR, et al. Socioeconomic inequalities in health in 22 European countries. *New Engl J Med* 2008;358:2468–81.

3 Mackenbach JP, Huisman M, Andersen O, et al. Inequalities in lung cancer mortality by the educational level in 10 European populations. *Eur J Cancer* 2004;40:126–35.

4 Adler NE, Boyce T, Chesney MA, et al. Socioeconomic status and health: the challenge of the gradient. *Am. Psychol.* 1993;49:15–24.

5 Braveman PA, Cubbin C, Egerter S, et al. Socioeconomic disparities in health in the United States: What the patterns tell us. *Am J Public Health*, 2010;(Suppl 1),186–96

6 Stringhini S, Batty GD, Bovet , et al. Association of Lifecourse Socioeconomic Status with Chronic Inflammation and Type 2 Diabetes Risk: The Whitehall II Prospective Cohort Study. *PLoS Med* 2013

7 Galobardes B, Shaw M, Lawlor DA, et al. Indicators of socioeconomic position (part 1). Journal of Epidemiology & Community Health 2006;60:7-12.

8 Kuh D, Ben-Shlomo Y, Lynch J, Hallqvist J, Power C. Life course epidemiology. J Epidemiol Community Health. 2005;57:778–83

9 McEwen BS, Gianaros PJ. Central role of the brain in stress and adaptation: Links to socioeconomic status, health, and disease, *Ann N Y Acad Sci*. 2010;1186:190–222.

10 Richard PS, Huang M-H, Sidney S, et. al. Socioeconomic status and health: is parasympathetic nervous system activity an intervening mechanism? *Int J Epid* 2005;34:309–315

11 Fox AJ, Goldblatt PO, Jones DR. Social class mortality differentials: artefact, selection, or life circumstance. *J. Epidemiol. Commun. Health* 198; 39: 1–8.

12 Mulatu MS, Schooler C. Causal connections between socio-economic status and health: reciprocal effects and mediating mechanisms *J. Health Soc. Behav.* 2002;43: 22-41

13 Galobardes B, Lynch J W, Smith G D. Childhood Socioeconomic Circumstances and Cause-specific Mortality in Adulthood: Systematic Review and Interpretation, *Epidemiologic Reviews*, Volume 26, Issue 1, 1 July 2004, Pages 7–21

14 Mayer KU. New directions in life course research. *Ann Rev Sociology 2009*;35:413–433.

15 Laurie M, Corna A. life course perspective on socioeconomic inequalities in health: A critical review of conceptual frameworks, *Adv Life Course Res*. 2013,18:150–159

16 Cutler DM, Lleras-Muney A, Vogl T. Socioeconomic Status and Health: Dimensions and Mechanisms. Publication Date: Sep 2012.

17 Hallqvist J, Lynch J, Bartley M, Lang T, Blane D. Can we disentangle life course processes of accumulation, critical period and social mobility? An analysis of disadvantaged socio-economic positions and myocardial infarction in the Stockholm Heart Epidemiology Program. Soc Sci Med. 2004;58:1555–62

For peer review only - http://bmjopen.bmj.com/site/about/guidelines.xhtml

18 Marin TJ, Chen E, Miller GE. What Do Trajectories of Childhood Socioeconomic Status Tell Us About Markers of Cardiovascular Health in Adolescence? *Psychosom Med* 2008;70:152–159

19 Matthews KA, Kiefe CI, Lewis CE, et al. Socioeconomic Trajectories and Incident Hypertension in a Biracial Cohort of Young Adults*. Hypertension* 2002;39:772-776.

20 Kozyrskyj AL, Kendall GE, Jacoby P, Sly PD, et al. Association Between Socioeconomic Status and the Development of Asthma: Analyses of Income Trajectories. *Am J Public Health*. 2010;100:540–546.

21 Tabak C, Spijkerman AMW, Verschuren WMM, et al. Does educational level influence lung function decline (Doetinchem Cohort Study)? *Eur Respir J* 2009; 34:940–947

22 Schünemann HJ, Dorn J, Grant BJB, et al. Pulmonary Function Is a Long-term Predictor of Mortality in the General Population*: 29-Year Follow-up of the Buffalo Health Study *Chest* 2000;118(3):656-664.

23 Stavem K, Aaser E, Sandvik L, et al. Lung function, smoking and mortality in a 26-year follow-up of healthy middle-aged Males. *Eur Respir J* 2005; 25 618–625

24 Johannessen A, Eagan TML, Omenaas ER, et al. Socioeconomic risk factors for lung function decline in a general population A. *Eur Respir J* 2010;36:480–487

25 Peasey A, Bobak M, Kubinova R, et al. Determinants of cardiovascular disease and other non-communicable disease in Central and Eastern Europe: rationale and design of the HAPIEE study. BMC Public Health. 2006;6:255.

26 Miller MR, Hankinsosn J, Brusasco V, et al. Standardisation of spirometry. *Eur. Respir. J. 2005* 26;319-338.

27 Vandenheede H, Vikhireva O, Pikhart H, et al. Socioeconomic inequalities in all-cause mortality in the Czech Republic, Russia, Poland and Lithuania in the 2000s: findings from the HAPIEE Study. J Epidemiol Community Health 2014;68:297-303.

28 Ciu T, Fang D, Chen J, and Jerois , C. (2001). A Robust and Scalable Clustering Algorithm, for Mixed Type Attributes in Large Database Environment. *In: Proceedings of the seventh ACM SIGKDD international conference on knowledge discovery and data mining. San Francisco, CA: ACM 2001*.

29 Gray LA, Leyland AH, Benzeval M, et al. Explaining the social patterning of lung function in adulthood at different ages: the roles of childhood precursors, health behaviours and environmental factors. *J Epidemiol Community Health*. 2013;67(11):905–11.

30 McFadden E, Luben R, Wareham N, et al. How far can we explain the social class differential in respiratory function? A cross-sectional population study of 21,991 men and women from EPIC-Norfolk. *Eur J Epidemiol*. 2009;24(4):193–201

31 Merkus PJ, ten Have-Opbroek AA, Quanjer PH Human lung growth: areview. *Pediatr Pulmonol* 1996, 21(6):383-397.

32 Hegewald MJ, Crapo RO. Socioeconomic status and lung function. *Chest*. 2007;132(5):1608–14.

33 Gaffney AW, Hang J, Lee MS, et al. Socioeconomic status is associated with reduce lung function in China: an analysis from large cross-sectional study in Shanghai. *BMC Public Health* 2016;16:96

34 Ramsay SE, Whincup PH, Lennon LT, et al. Longitudinal associations of socioeconomic position in childhood and adulthood with decline in lung function over 20 years: results from a population-based cohort of British men. *Thorax.* 2011 Dec;66(12):1058-64.

35 Conry MC, Morgan K, Curry P, McGee H, Harrington J, Ward M, et al. The clustering of health behaviours in Ireland and their relationship with mental health, self-rated health and quality of life. BMC Public Health. 2011;11(1):692–701.

For peer review only - http://bmjopen.bmj.com/site/about/guidelines.xhtml

36 Clatworthy J, Hankins M, Buick D, et al. Cluster analysis in illness perception research: A Monte Carlo study to identify the most appropriate method. Psychology & Health 2007; 22:2,123-142,

37 Krzyzanowski M, Jedrychowski W. Occupational exposure and incidence of chronic respiratory symptoms among residents of Cracow followed for 13 years. Int Arch Occup Environ Health. 1990;62(4):311-7

38 Przewozniak K. , Sulkowska U, et al.. Tobacco smoking in countries of the European Union. Ann Agric Environ Med. 2012;19(2):181-92.

39 Menezes MB, Dumith SC, Perez-Padilla R, et al. Socioeconomic trajectory from birth to adolescence and lung function: prospective birth cohort study. *BMC Public Health*;2011,11:596

40 Shaheen SO, Jameson KA, Syddall HE, et al. The relationship of dietary patterns with adult lung function and COPD. *Eur Respir J.* 2010;36(2):277–84.

41Tabak C, Smit HA, Rasanen L, et al. Dietary factors and pulmonary function: a cross sectional study in middle aged men from three European countries. *Thorax*. 1999;54(11):1021–6.

42 Strachan DP. Ventilatory function, height, and mortality among lifelong non-smokers. *J Epidemiol Community Health* 1992; 46:66–70.

43 Batty GD, Lawlor DA, Macintyre S, et al. Accuracy of adults' recall of childhood social class: findings from the Aberdeen children of the 1950s study. *J Epidemiol Community Health* 2005;59:898-903.

For peer review only

For peer review only - http://bmjopen.bmj.com/site/about/guidelines.xhtml

STROBE Statement—checklist of items that should be included in reports of observational studies

	Item No	Recommendation
**Title and abstract**	1	Indicate the study's design with a commonly used term in the title or the abstract – page 1, lines: 2-3
		(*b*) Provide in the abstract an informative and balanced summary of what was done and what was found – page 2, lines :11-25
**Introduction**		
Background/rationale	2	Explain the scientific background and rationale for the investigation being reported – page 4, lines 2-8; page 5, lines: 12-18
Objectives	3	State specific objectives, including any prespecified hypotheses page 5, lines:12-18
**Methods**		
Study design	4	Present key elements of study design early in the paper – page 6, lines3-4
Setting	5	Describe the setting, locations, and relevant dates, including periods of recruitment, exposure, follow-up, and data collection – page 6, lines:5-9
Participants	6	(*a*) *Cohort study*—Give the eligibility criteria, and the sources and methods of selection of participants. Describe methods of follow-up *Case-control study*—Give the eligibility criteria, and the sources and methods of case ascertainment and control selection. Give the rationale for the choice of cases and controls *Cross-sectional study*—Give the eligibility criteria, and the sources and methods of selection of participants – page 6, lines: 5-8
		(*b*) *Cohort study*—For matched studies, give matching criteria and number of exposed and unexposed *Case-control study*—For matched studies, give matching criteria and the number of controls per case
Variables	7	Clearly define all outcomes, exposures, predictors, potential confounders, and effect modifiers. Give diagnostic criteria, if applicable – page 6, lines:24-31, pages 7-8
Data sources/ measurement	8*	For each variable of interest, give sources of data and details of methods of assessment (measurement). Describe comparability of assessment methods if there is more than one group – pages 6-9
Bias	9	Describe any efforts to address potential sources of bias page – page 17, lines 26-28
Study size	10	Explain how the study size was arrived at – page 10, lines:6-9
Quantitative variables	11	Explain how quantitative variables were handled in the analyses. If applicable, describe which groupings were chosen and why – page 9, lines: 13-14
Statistical methods	12	(*a*) Describe all statistical methods, including those used to control for confounding– pages 9, lines: 13-31
		(*b*) Describe any methods used to examine subgroups and interactions – page 9, lines: 25-30
		(*c*) Explain how missing data were addressed– page 10, lines:6-7
		(*d*) *Cohort study*—If applicable, explain how loss to follow-up was addressed *Case-control study*—If applicable, explain how matching of cases and controls was addressed *Cross-sectional study*—If applicable, describe analytical methods taking account of sampling strategy
		(*e*) Describe any sensitivity analyses

For peer review only

**Results**		
Participants	13*	(a) Report numbers of individuals at each stage of study—eg numbers potentially eligible, examined for eligibility, confirmed eligible, included in the study, completing follow-up, and analysed – page 10, lines: 6-9
		(b) Give reasons for non-participation at each stage
		(c) Consider use of a flow diagram
Descriptive data	14*	(a) Give characteristics of study participants (eg demographic, clinical, social) and information on exposures and potential confounders – page 10, lines,; page 11, lines: 1-7
		(b) Indicate number of participants with missing data for each variable of interest – page 10, lines: 6-8
		(c) *Cohort study*—Summarise follow-up time (eg, average and total amount)
Outcome data	15*	*Cohort study*—Report numbers of outcome events or summary measures over time
		Case-control study—Report numbers in each exposure category, or summary measures of exposure
		Cross-sectional study—Report numbers of outcome events or summary measures – page 11, table
Main results	16	(*a*) Give unadjusted estimates and, if applicable, confounder-adjusted estimates and their precision (eg, 95% confidence interval). Make clear which confounders were adjusted for and why they were included – pages 12-13
		(*b*) Report category boundaries when continuous variables were categorized – not applicable
		(*c*) If relevant, consider translating estimates of relative risk into absolute risk for a meaningful time period
Other analyses	17	Report other analyses done—eg analyses of subgroups and interactions, and sensitivity analyses – page 10, lines 7-11
**Discussion**		
Key results	18	Summarise key results with reference to study objectives – page 15, lines 2-5
Limitations	19	Discuss limitations of the study, taking into account sources of potential bias or imprecision. Discuss both direction and magnitude of any potential bias – page 17, lines 20-28
Interpretation	20	Give a cautious overall interpretation of results considering objectives, limitations, multiplicity of analyses, results from similar studies, and other relevant evidence – page 14
Generalisability	21	Discuss the generalisability (external validity) of the study results– page 17, lines 7-19
**Other information**		
Funding	22	Give the source of funding and the role of the funders for the present study and, if applicable, for the original study on which the present article is based– page 19

*Give information separately for cases and controls in case-control studies and, if applicable, for exposed and unexposed groups in cohort and cross-sectional studies.

Note: An Explanation and Elaboration article discusses each checklist item and gives methodological background and published examples of transparent reporting. The STROBE checklist is best used in conjunction with this article (freely available on the Web sites of PLoS Medicine at http://www.plosmedicine.org/, Annals of Internal Medicine at http://www.annals.org/, and Epidemiology at http://www.epidem.com/). Information on the STROBE Initiative is available at www.strobe-statement.org.

For peer review only - http://bmjopen.bmj.com/site/about/guidelines.xhtml
